# Individual liver plasmacytoid dendritic cells are capable of producing IFNα and multiple additional cytokines during chronic HCV infection

**DOI:** 10.1371/journal.ppat.1007935

**Published:** 2019-07-29

**Authors:** Erin Heather Doyle, Adeeb Rahman, Costica Aloman, Arielle L. Klepper, Ahmed El-Shamy, Francis Eng, Chiara Rocha, Sang Kim, Brandy Haydel, Sander S. Florman, M. Isabel Fiel, Thomas Schiano, Andrea D. Branch

**Affiliations:** 1 Division of Liver Diseases, Icahn School of Medicine at Mount Sinai, New York, New York, United States of America; 2 Human Immune Monitoring Core, Icahn School of Medicine at Mount Sinai, New York, New York, United States of America; 3 Rush University Medical Center, Chicago, Illinois, United States of America; 4 Recanati Miller Transplantation Institute, The Mount Sinai Hospital, New York, New York, United States of America; 5 Department of Anesthesiology, The Mount Sinai Hospital, New York, New York, United States of America; 6 Department of Pathology, The Mount Sinai Hospital, New York, New York, United States of America; The University of Chicago, UNITED STATES

## Abstract

Plasmacytoid dendritic cells (pDCs) are “natural” interferon α (IFNα)-producing cells. Despite their importance to antiviral defense, autoimmunity, and ischemic liver graft injury, because DC subsets are rare and heterogeneous, basic questions about liver pDC function and capacity to make cytokines remain unanswered. Previous investigations failed to consistently detect IFNα mRNA in HCV-infected livers, suggesting that pDCs may be incapable of producing IFNα. We used a combination of molecular, biochemical, cytometric, and high-dimensional techniques to analyze DC frequencies/functions in liver and peripheral blood mononuclear cells (PBMCs) of hepatitis C virus (HCV)-infected patients, to examine correlations between DC function and gene expression of matched whole liver tissue and liver mononuclear cells (LMCs), and to determine if pDCs can produce multiple cytokines. T cells often produce multiple cytokines/chemokines but until recently technical limitations have precluded tests of polyfunctionality in individual pDCs. Mass cytometry (CyTOF) revealed that liver pDCs are the only LMC that produces detectable amounts of IFNα in response TLR-7/8 stimulation. Liver pDCs secreted large quantities of IFNα (~2 million molecules of IFNα/cell/hour) and produced more IFNα than PBMCs after stimulation, p = 0.0001. LMCs secreted >14-fold more IFNα than IFNλ in 4 hours. Liver pDC frequency positively correlated with whole liver expression of “IFNα-response” pathway (R^2^ = 0.58, p = 0.007) and “monocyte surface” signature (R^2^ = 0.54, p = 0.01). Mass cytometry revealed that IFNα-producing pDCs were highly polyfunctional; >90% also made 2–4 additional cytokines/chemokines of our test set of 10. Liver BDCA1 DCs, but not BDCA3 DCs, were similarly polyfunctional. pDCs from a healthy liver were also polyfunctional. Our data show that liver pDCs retain the ability to make abundant IFNα during chronic HCV infection and produce many other immune modulators. Polyfunctional liver pDCs are likely to be key drivers of inflammation and immune activation during chronic HCV infection.

## Author summary

This is a detailed characterization of human liver plasmacytoid dendritic cells from patients with a chronic viral infection. It revealed that these rare innate immune cells can become point sources of multiple immune activators and pro-inflammatory mediators. This study adds new information about the fundamental properties of pDCs, which are traditionally known as “natural interferon producing cells.” In fact, these cells produce an array of bioactive molecules and may play an important role in organizing the liver’s immune response.

## Introduction

Plasmacytoid dendritic cells (pDCs) are rare innate immune cells that comprise about 0.5% of peripheral blood mononuclear cells (PBMCs). They migrate into tissues and are known as “natural” producers of interferon alpha (IFNα). pDCs constitutively express toll-like receptor (TLR)-7 and TLR-9, as well as interferon regulatory factor (IRF)-7, enabling them to detect viral nucleic acids and to quickly secrete type I IFNs (IFNα and IFNβ), which bind neighboring cells and induce hundreds of IFN stimulated genes (ISGs), initiating antiviral defenses.

The activity of pDCs during HCV infection remains obscure. Several groups examined pDC frequency and function during chronic infection. Nearly all found a reduced frequency of pDCs in blood [1–7]. Some reported that circulating pDCs are functionally intact [6, 7], but the majority reported impairment after stimulation with various TLR ligands [1–5], which was attributed to toxic effects of tumor necrosis factor α (TNFα) [5] and direct inhibitory effects of HCV proteins [8] [9]. In contrast to these inhibitory effects, HCV RNA stimulates pDCs by activating TLR-7 and RIG-I [10–13].

Past studies of patients and chimpanzees provide circumstantial evidence that liver pDCs do not produce IFNα during HCV infection. *IFNA* mRNA levels are not consistently elevated during acute [14] or chronic [15, 16] infection, and liver *IFNA2* mRNA levels rose to detectable levels only after HCV was cured [17], suggesting that HCV may shut down IFNα production. The absence of detectable *IFNA* mRNA was initially puzzling because ISGs are highly induced in HCV-infected liver [18], but the discovery of type III IFNs (IFNλs) provided a possible explanation for the seeming paradox because these cytokines up-regulate many of the same genes as IFNα [19]. These investigations left the question of pDC functionality during HCV infection unanswered.

We explored an alternative explanation: the possibility that intrahepatic pDCs remain functional during chronic HCV infection but generate an *IFNA* mRNA signal that is too low to be detected in extracts of whole liver tissue. To improve the signal-to-noise ratio, liver mononuclear cells (LMCs) were purified and examined in parallel with whole liver tissue and PBMCs. We found that liver pDCs retain the ability to produce large quantities of IFNα and made more IFNα per cell than blood pDCs. Liver pDC frequency had strong positive correlations with whole liver expression of the “IFNα-response (I)” pathway of blood transcriptomic (BT) modules and with three monocyte-specific modules, indicating that pDCs are active *in vivo* and have effects on other liver immune cells. Single-cell mass cytometry (CyTOF) revealed that liver pDCs are the only LMCs that make IFNα and demonstrated that most IFNα-producing pDCs are polyfunctional and a single IFNα^+^ pDC makes several additional cytokines/chemokines. Individual liver BDCA1 DCs were similarly polyfunctional. These findings demonstrate that intrahepatic pDCs and BDCA1 DCs can be intense point sources of a constellation of immune activators and they establish that liver pDCs remain competent for IFNα production despite chronic exposure to viral products.

## Results

### Experimental design and characteristics of the study subjects

**Fig 1:**
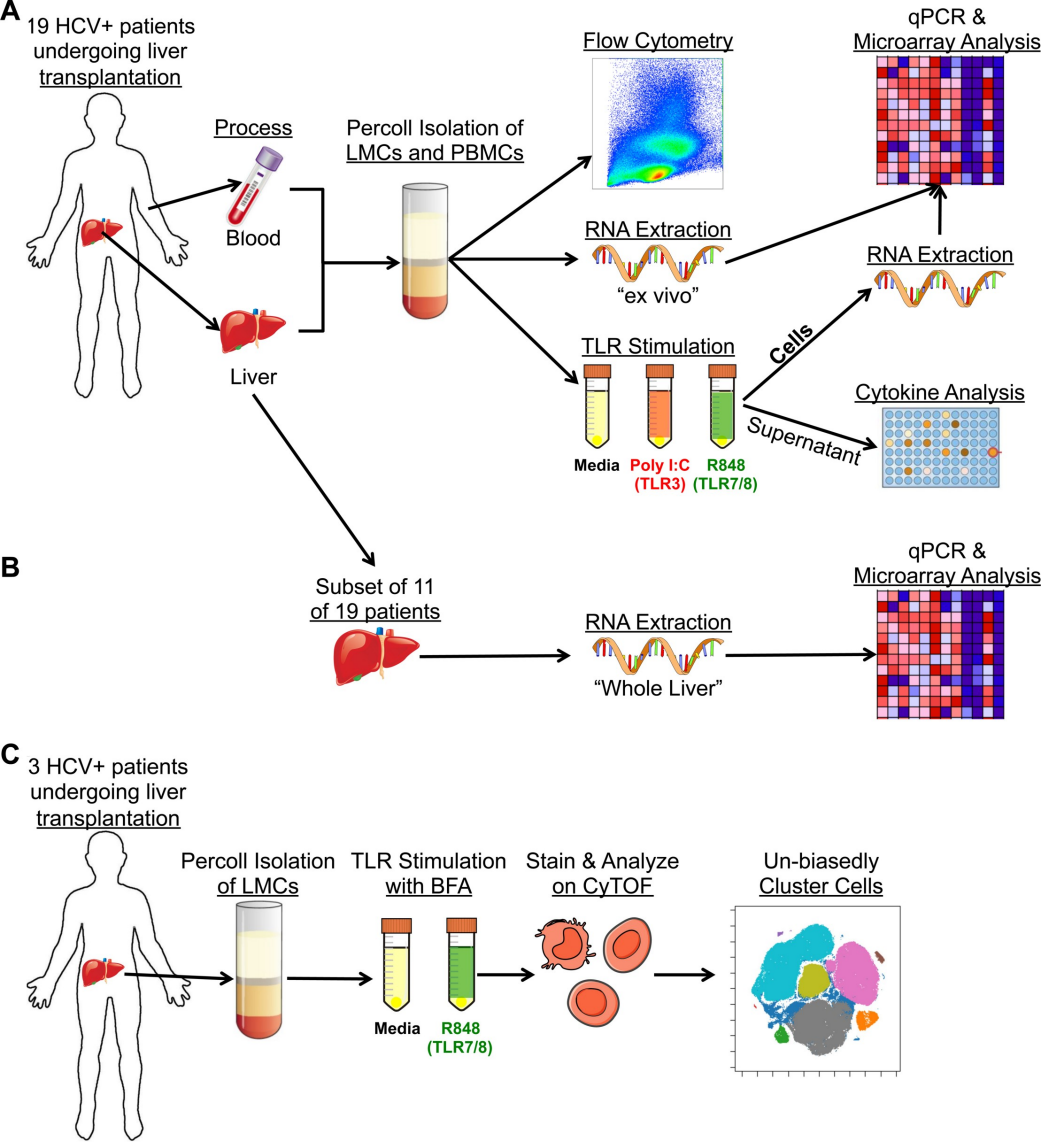
Workflow of analysis. (A) Matched blood and liver were obtained from 19 HCV^+^ patients. LMCs and PBMCs were isolated and analyzed “ex vivo” by flow cytometry and RNA extraction followed by RT/qPCR and microarray analysis, or they were stimulated with TLR3 or TLR-7/8 agonists followed by cytokine or RNA analysis. (B) RNA was extracted from total liver of 11 patients then analyzed by RT/qPCR and microarray. (C) LMCs were obtained from three additional patients, stimulated with a TLR7/8 agonist in the presence of BFA, and analyzed by CyTOF.

Medical record data, liver, and blood were obtained from 19 patients with chronic HCV infection who were undergoing liver transplantation. The median age was 62 years [interquartile range (IQR), 59–65]; 79% were male (S1 Table). The median natural model for end stage liver disease (MELD), which assesses the amount of liver damage, was 18 (IQR, 13–32). LMCs and PBMCs were prepared by density gradient centrifugation and either examined immediately, “*ex vivo*”, by flow cytometry, microarray, and RT/PCR or they were incubated for four hours with TLR ligands (or media alone) prior to analysis (Fig 1A). Total *ex vivo* liver mRNA of 11 of the 19 patients was analyzed by microarray and RT/qPCR (Fig 1B). LMCs of three additional anonymous HCV^+^ patients were analyzed by CyTOF (Fig 1C).

### *In vivo* activity of liver pDCs

**Fig 2:**
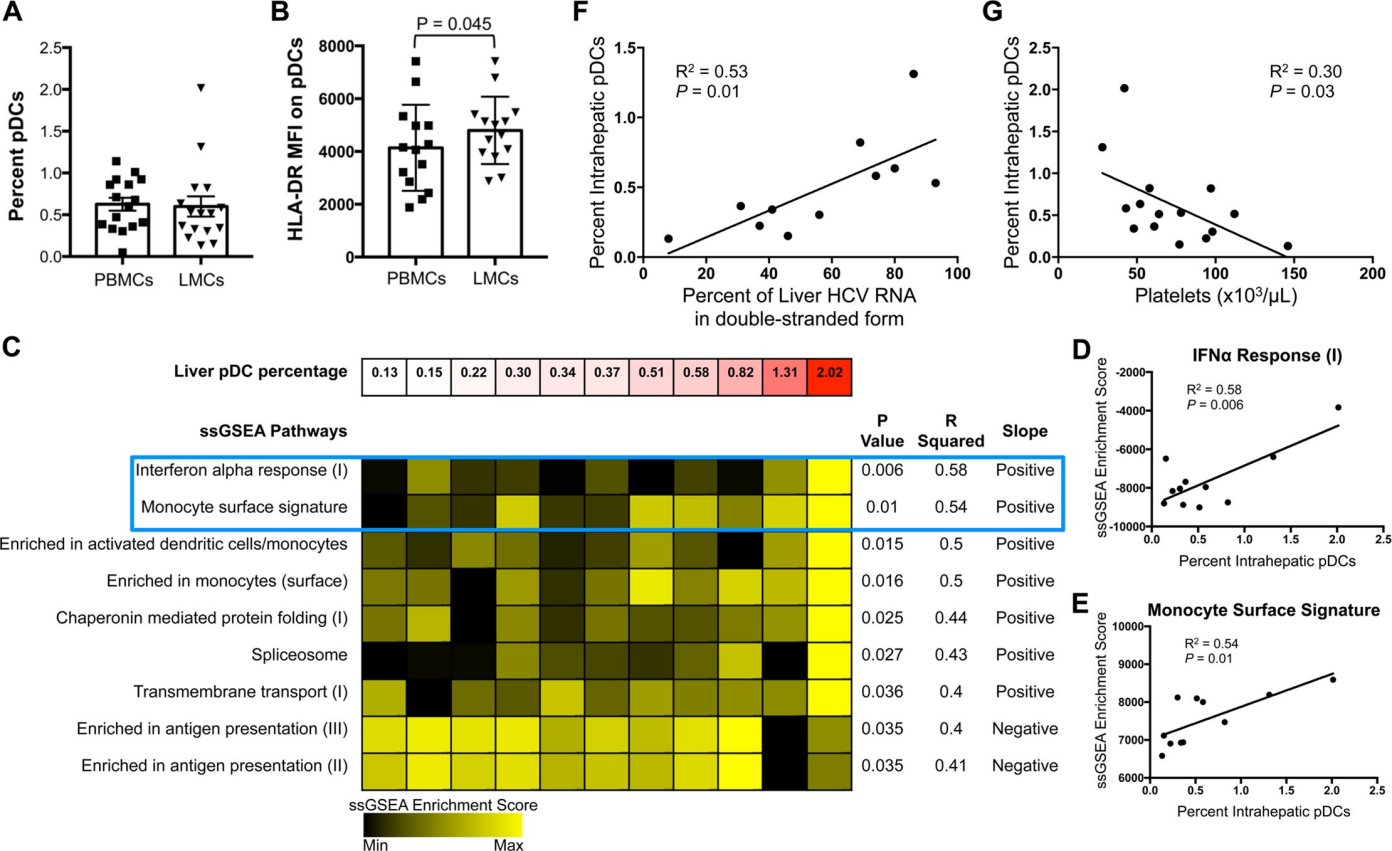
pDCs do not concentrate in the liver and are associated with liver ISG response. (A) Percentage pDCs of total CD45^+^ LMCs and PBMCs. (B) MFI of HLA-DR on pDCs from blood and liver. Horizontal bars depict the mean±SD; N = 16, paired *t* test. (C) Pathways from ssGSEA analysis of microarray data of whole liver tissue that correlated with liver pDC frequency. Each column represents ssGSEA enrichment score of one individual (N = 11, Pearson’s correlation coefficient). Examples of the two top scatter plots are encompassed in blue box and in D and E. (D) Scatter plot of “Interferon α (IFNα) Response” pathway from C. (E) Scatter plot of “Monocyte Surface Signature” pathway from C. (F) Correlation between liver pDC frequency and percent intrahepatic HCV RNA in double-stranded form or (G) serum platelets (N = 16, Pearson’s correlation coefficient).

pDC frequencies in CD45^+^ PBMCs and LMCs were determined by flow cytometry using the gating strategy in S1 Fig, although this gating strategy does not rule out the possibility of including preDCs within the pDC population [20]. The pDC frequency was the same in liver and blood, suggesting that pDCs do not concentrate in the liver (Fig 2A), but the median fluorescent intensity (MFI) of HLA-DR on the liver pDCs was higher (Fig 2B), indicating greater activation. The impact of pDCs on surrounding liver cells was analyzed by examining correlations between pDC frequency and transcriptomic data of 11 whole livers. Four modules had a strong correlation (R^2^≥0.5) with liver pDC frequency (Fig 2C–2E): “IFNα response (I)” (Fig 2D), “Monocyte surface signature” (Fig 2E), “Enriched in activated dendritic cells/monocytes,” and “Enriched in monocytes (surface).” These results indicate that liver pDCs are active *in vivo*. Fifty-four percent of the genes in these four blood transcription (BT) modules are part of the Interferome [21]. The genes in these and other BT modules are listed in S4 Table. Liver pDC frequency also strongly correlated with the percentage of liver HCV RNA in double-stranded form (Fig 2F), consistent with published data showing that IFNα increases HCV RNA duplexes [22]. Analysis of clinical data revealed a significant inverse relationship between liver pDC frequency and blood platelet counts (p = 0.03, Fig 2G).

### BDCA1^+^ and BDCA3^+^ DC subsets in blood and liver

**Fig 3:**
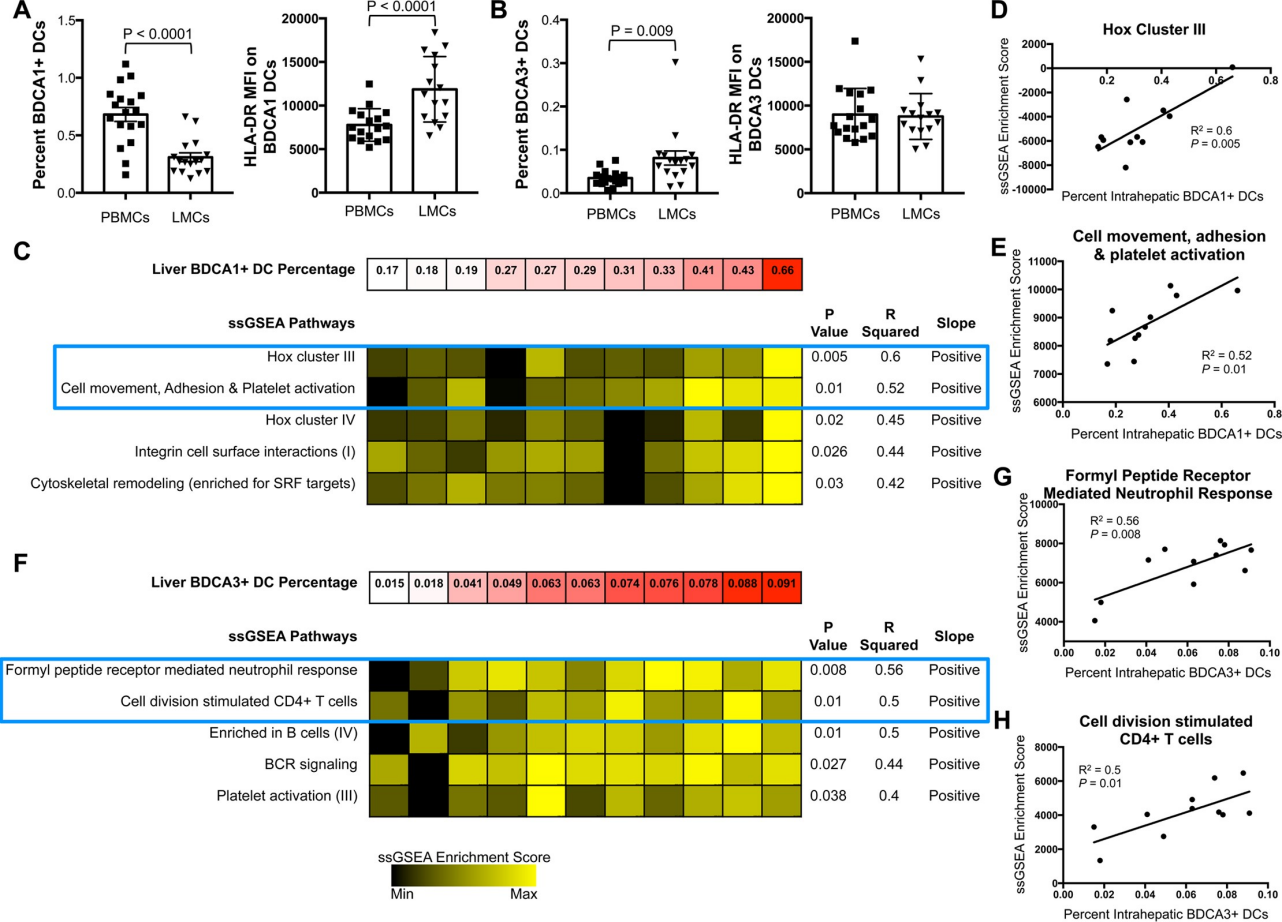
Analysis of BDCA1^+^ and BDCA3^+^ DCs. (A) Frequency of BDCA1^+^ DCs in LMCs/PBMCs (left) and MFI of HLA-DR on BDCA1^+^ DCs (right). (B) Frequency of BDCA3^+^ DCs in LMCs/PBMCs (left) and MFI of HLA-DR on BDCA3^+^ DCs (right). Horizontal bars depict mean±SD. N = 16, paired *t* test. (C) Top pathways from ssGSEA analysis of microarray data isolated from whole liver tissue that correlated with percent of intrahepatic BDCA1^+^ DCs. Examples of two top scatter plots are encompassed in blue box and in D and E. (D) Scatter plot of “Hox Cluster III” pathway from C. (E) Scatter plot of “Cell movement, adhesion, and platelet activation” pathway from C. (F) Top pathways from ssGSEA analysis of microarray data isolated from whole liver tissue that correlated with percentage of intrahepatic BDCA3^+^ DCs. Examples of two top scatter plots are encompassed in blue box and in G and H. (G) Scatter plot of “Formyl Peptide Receptor Mediated Neutrophil Response” pathway from F. (H) Scatter plot of “Cell division stimulated CD4+ T cells” pathway from F. N = 11, Pearson’s correlation coefficient.

We also analyzed two additional DC subsets, BDCA1 and BDCA3 DCs. As determined by flow cytometry, BDCA1^+^ (classical) DCs were enriched in blood compared to liver (Fig 3A, left), while BDCA3^+^ (cross-presenting) DCs were enriched in liver (Fig 3B, left). The MFI of HLA-DR was higher on liver BDCA1^+^ DCs than on their counterparts in blood (Fig 3A, right), but HLA-DR did not differ between liver and blood BDCA3^+^ DCs (Fig 3B, right).

Single sample gene set enrichment analysis (ssGSEA) revealed a strong correlation between the frequency of intrahepatic BDCA1^+^ DCs and expression of “Hox cluster III”, R^2^ = 0.6 (Fig 3C and 3D) and “Cell movement, Adhesion & Platelet activation”, R^2^ = 0.52 (Fig 3C and 3E). Hox genes are critical for proliferation and differentiation of hematopoietic cells, especially T cells [23]. Liver BDCA3^+^ frequency strongly correlated with three pathways (Fig 3F): “Formyl peptide mediated neutrophil response” (Fig 3G), “Cell division stimulated CD4^+^ T cells” (Fig 3H), and “Enriched in B cells (IV).” Taken together, these data suggest that BDCA1^+^ and BDCA3^+^ DCs increase immune infiltration, migration, and induction of adaptive and innate immune responses.

### pDCs are the only LMC subset that produce IFNα

**Fig 4:**
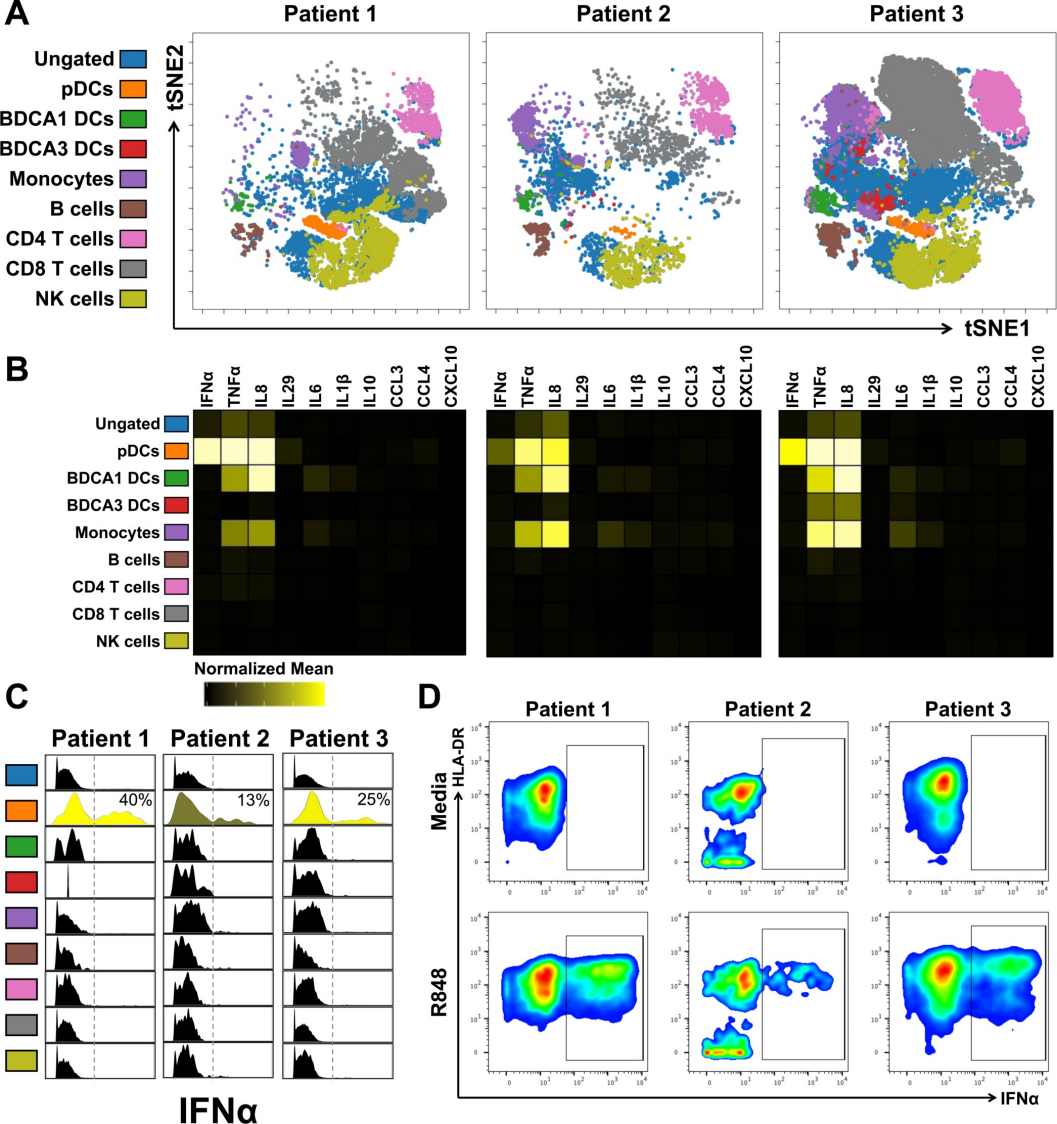
IFNα was made exclusively by pDCs. (A) Unbiased viSNE plot of CyTOF data from R848-stimulated LMCs of 3 HCV^+^ individuals. Major immune subsets were manually gated and mapped onto viSNE plot. (B) Heatmap of normalized mean signal intensity (nMSI) of all cytokines/chemokines for each immune subset. (C) Histogram of signal intensity of IFNα for each immune subset; insert is percent pDCs expressing IFNα. (D) Manual gating of IFNα^+^ pDCs for 3 HCV^+^ individuals both with and without stimulation.

CyTOF was used to definitively identify the IFNα-producing liver cells (Fig 1C); the CyTOF antibody panel is presented in S5 Table. LMCs were analyzed after incubation for 4 hours with media or R848, a TLR7/8 agonist, in the presence of brefeldin A (BFA) to block cytokine secretion. viSNE was employed to project the high-dimensional data onto two-dimensional space. Nine major subsets of CD45^+^ cells were identified based on canonical markers (Fig 4A; S1 Fig). pDCs comprised a distinct cluster in all three patients (Fig 4A, orange). The normalized mean signal intensity (nMSI) for IFNα (Fig 4B) was determined for all subsets and IFNα positivity was plotted for each population (Fig 4C). pDCs comprised the only population of IFNα^+^ cells (Fig 4B and 4C); on average 26% (13–40%) of the pDCs expressed IFNα following R848 stimulation (Fig 4C and 4D).

### IFNα and IFNλ production by LMCs and PBMCs

**Fig 5:**
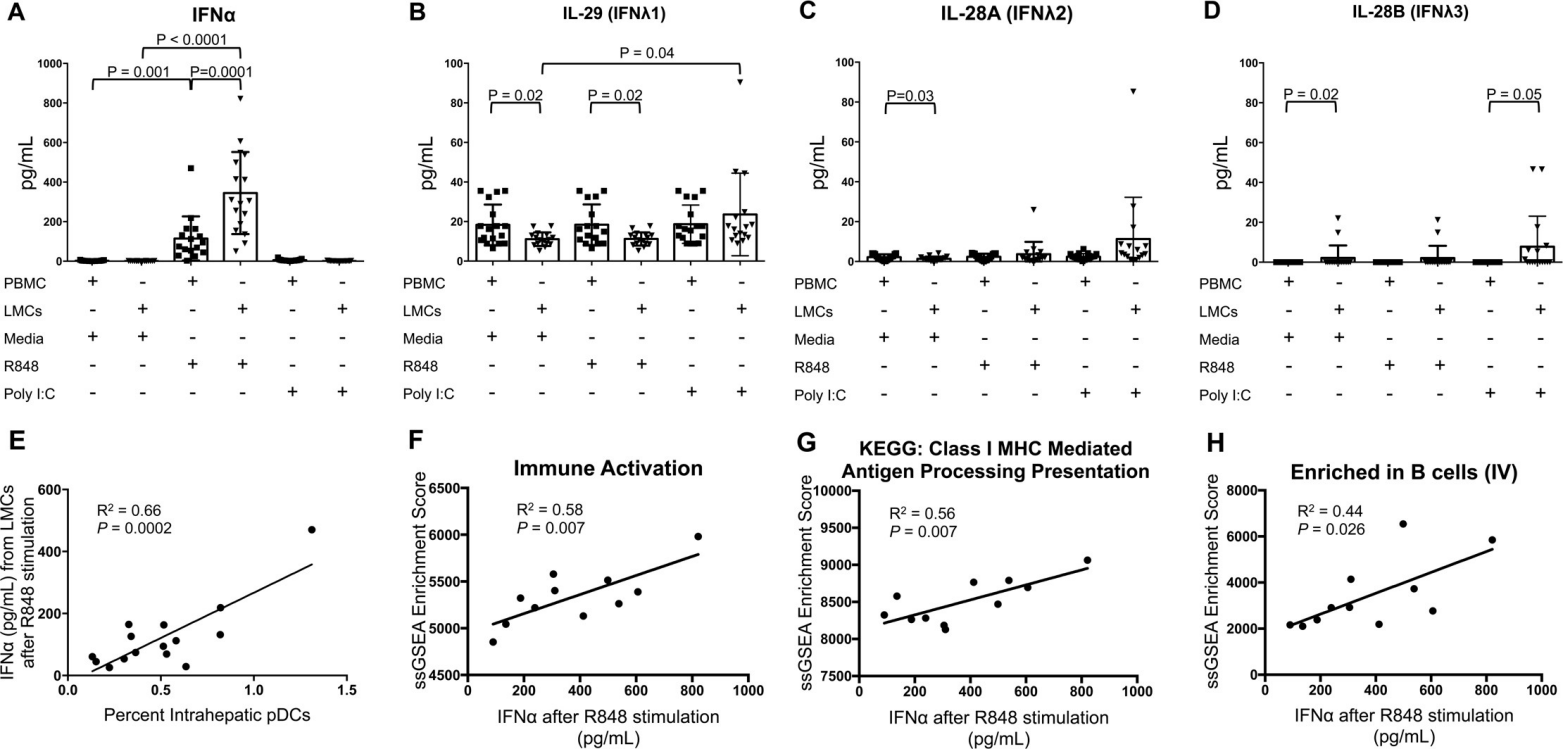
IFNα secretion and relationship with immune activation in whole liver. LMCs and matched PBMCs incubated in R848, PolyI:C or media for 4 hr and secretion (pg/mL) of (A) IFNα, (B) IL-29 (IFNλ1), (C) IL-28A (IFNλ2), and (D) IL-28B (IFNλ3) were measured. Horizontal bars depict mean±SD. N = 17, paired t-tests. (E) Correlation between amount of IFNα secreted by R848-stimulated LMCs and percentage of intrahepatic pDCs (N = 15, Pearson’s correlation coefficient). (F–H) Pathways from ssGSEA analysis of microarray data of whole liver tissue that significantly correlated with the amount of IFNα secreted by R848-stimulated LMCs (N = 11, Pearson’s correlation coefficient); (F) scatter plot of “Immune Activation–generic cluster” pathway, (G) “KEGG: Class I MHC Mediated Antigen Processing Presentation” pathway, and (H) “Enriched in B cells (IV)” pathway are shown.

The quantity of IFNs secreted by liver pDCs and other LMCs was investigated (Fig 5A–5D) after incubation in media alone, with R848 or with Poly I:C, a TLR-3 agonist that is important for IFNλ production [24]. LMCs secreted 3-fold more IFNα than PBMCs in response to R848 stimulation, 345±207 pg/mL vs. 115±111 pg/mL, p = 0.0001 (Fig 5A).

The amount of IFNα secreted per liver pDC per hour was calculated by combining data from flow cytometry, Luminex, and CyTOF. Secretion assays contained a mean of 6000 pDCs, with ~26% (1560 pDCs) producing IFNα. Mean secretion was 86 pg of IFNα2a/2b/hour, which indicates that each IFNα-producing pDC was secreting over 1.7x10^6^ molecules per hour. Actual secretion may exceed this number because the Luminex assay targets only IFNα2a/2b and there are 11 additional forms of human IFNα. Wimmers *et al*. showed that over the course of 12 hours, the small percentage of pDCs that initially produce IFNα later induce IFNα production in neighboring pDCs, in a local amplification loop [25]. Our calculation does not consider this amplification process because our incubations were only for 4 hours.

The amount of IFNα secreted by LMCs correlated strongly with the frequency of liver pDCs, p = 0.0002 (Fig 5E), consistent with CyTOF data indicating that pDCs are the only IFNα producers (Fig 4B). It also correlated with expression of the “Immune activation-generic cluster” in whole liver, p = 0.007 (Fig 5F), as well as expression of the KEGG pathway “Class I MHC Mediated Antigen Processing Presentation” (p = 0.007, Fig 5G) and “Enriched in B cells (IV)” (p = 0.026, Fig 5H). Minimal IFNα was secreted by LMCs or PBMCs incubated in media alone or with Poly I:C. Compared to IFNα, LMCs secreted far less IFNλ1 or 2/3. The greatest amount was 24±21 pg/mL of IFNλ1 (Fig 5B–5D), which is more than 14-fold *lower* than the greatest amount of IFNα. The quantities of IFNλ secreted by LMCs in response to TLR stimulation did not correlate with the frequency of any of the three DC subsets we examined.

**Fig 6:**
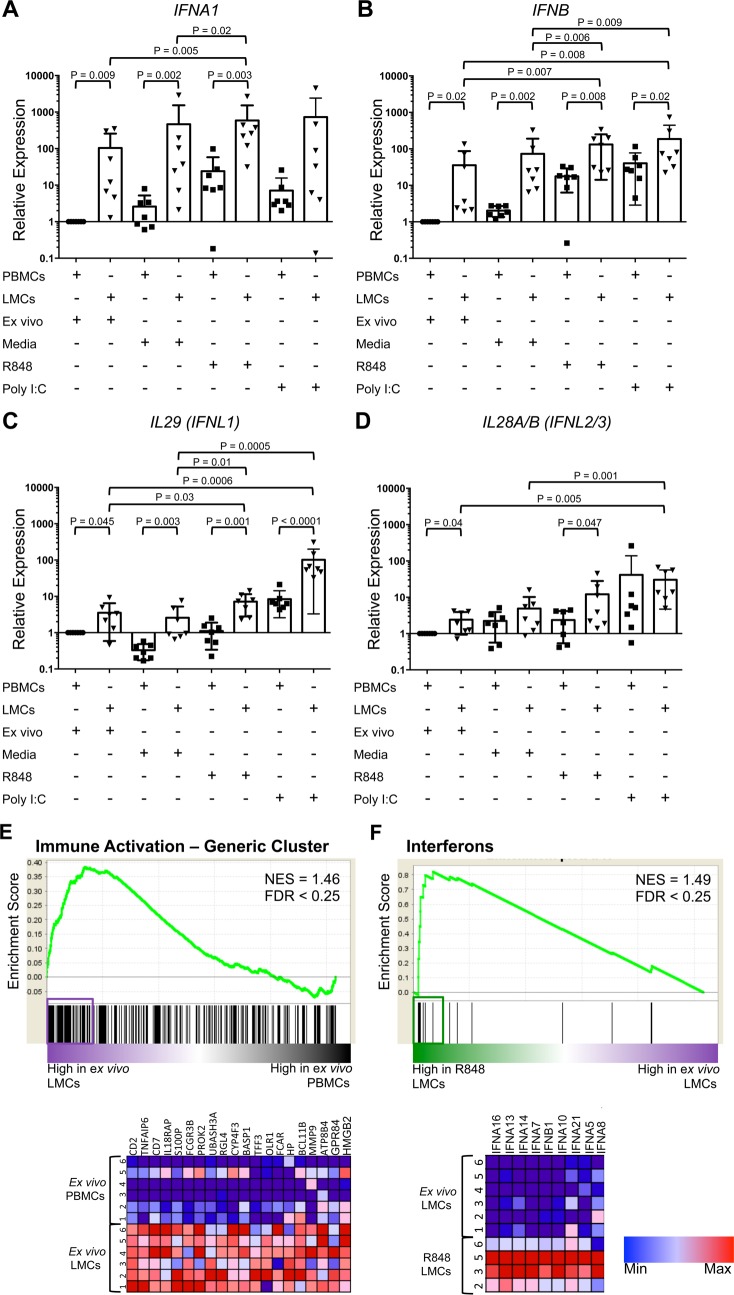
Message levels of type I and type III IFNs. Expression of (A) *IFNA1*, (B) *IFNB*, (C) *IL29 (IFNL1)*, and (D) *Il28A/B (IFNL2/3)* in LMCs and PBMCs either *ex vivo* or after incubation with media, PolyI:C, or R848, relative to *ex vivo* PBMCs. Horizontal bars depict mean±SD. N = 7, ratio paired t-test. (E) GSEA of “Immune Activation-Generic Cluster” comparing *ex vivo* LMCs to *ex vivo* PBMCs. (F) GSEA of “Interferons” comparing ex *vivo* LMCs and R848-stimulated LMCs. Pathways with a false discovery rate (FDR) of less than 0.25 were considered significant. Genes contributing to pathway enrichment (leading edge genes) are boxed and shown in heat map below.

RT/qPCR and microarrays were used to investigate gene expression in LMCs, PBMCs, and whole liver. Notably, *IFNA1* mRNA was readily detected in *ex vivo* LMCs by RT/qPCR (Fig 6A), but neither *IFNA1* mRNA, nor any of the other *IFN* mRNAs could be detected by RT/qPCR in whole liver: all 11 whole liver extracts had Ct values above 35. *Ex vivo* LMCs expressed higher levels of *IFNA1*, *IFNB*, and type III IFN mRNA (*IL29* and *IL28A/B*) than *ex vivo* PBMCs (Fig 6A–6D). Consistent with the RT/qPCR results, microarray data showed that *ex vivo* LMCs had higher expression of the “Immune Activation–Generic Cluster” and higher “TLR and Inflammatory Signaling” than PBMCs (Fig 6E and S2B Fig, respectively).

IFN gene expression was also examined following four hours of incubation in media with and without TLR ligands. RT/qPCR analysis showed that the TLR-7/8 agonist, R848, increased expression of *IFNA1* and *IFNB* in LMCs compared to *ex vivo* LMCs and compared to LMCs incubated in media (Fig 6A and 6B). R848 treatment also increased *IL29* expression in LMCs relative to *ex vivo* LMCs, but it did not increase *IL28A/B* expression (Fig 6C and 6D). GSEA of microarray data of LMCs showed that R848 treatment up-regulated many IFNα genes (Fig 6F) and enhanced the “antiviral IFN signature” (S2A Fig). The TLR-3 agonist, Poly I:C, did not induce *IFNA1* in LMCs, but it did induce *IFNB*, *IL29*, and *IL28A/B* (Fig 6A–6D). Poly I:C enhanced the “antiviral IFN signature” relative to *ex vivo* LMCs (S2C Fig), but not as intensely as R848 (S2D Fig). Compared to *ex vivo* or media, Poly I:C did not increase expression of any of the type I or type III IFN genes in PBMCs.

### Secretion of TNFα and additional cytokines

To explore the cytokine milieu more fully, TNFα, CXCL10, IL-6, and IL-10 secretion were examined by Luminex. LMCs made an average of 10-fold more TNFα (66±79 vs. 6±5 pg/mL), 3-fold more CXCL10 (278±236 vs. 98±110 pg/mL), 8-fold more IL-10 (16±12 vs. 2±2 pg/mL), and 10-fold more IL-6 (269±472 vs. 7±10 pg/mL) than PBMCs after incubation in media (*without* TLR stimulation), p≤0.05 for all comparisons (S3A–S3D Fig).

### Individual pDCs are point sources of IFNα and a constellation of additional cytokines and chemokines

**Fig 7:**
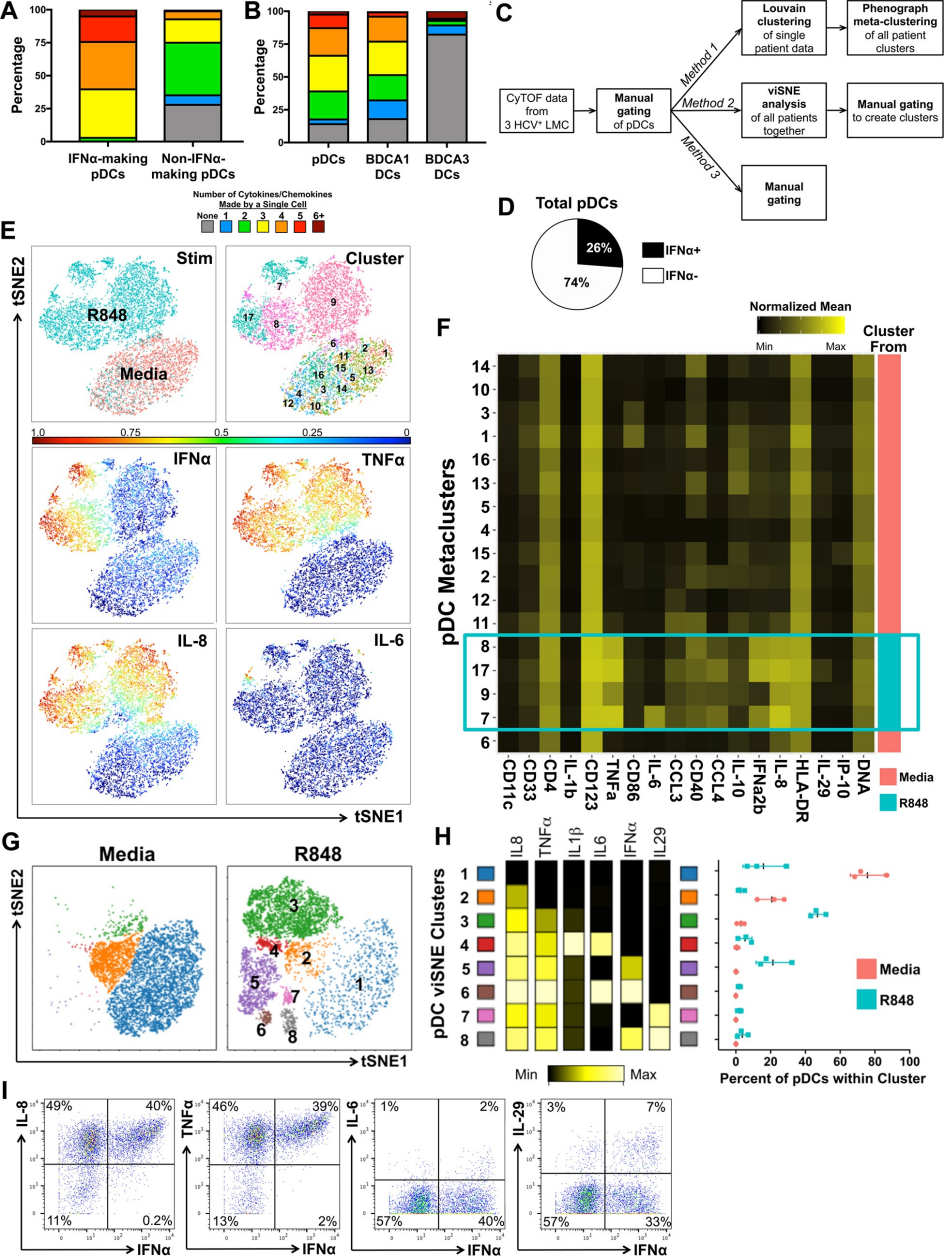
pDCs are highly polyfunctional. (A) Polyfunctional profiling of IFNα-producing pDCs and non-IFNα-producing pDCs. Each color represents the average number of cytokines/chemokines (of 10 in our panel) produced by a single pDC; IFNα-producing pDCs automatically start off producing 1 (or more) cytokine aka IFNα. (B) Polyfunctional profiling of all pDCs, BDCA1 DCs, and BDCA3 DCs. Each color represents the average number of cytokines/chemokines (of 10 in our panel) produced by a single cell. (C) Methods used to analyze CyTOF data. (D) Pie chart of the average IFNα+ pDC. (E) Gated pDCs were analyzed using Louvain clustering and Phenograph Metaclustering. Representative tSNE panels of Patient 1 showing which cells are from media- or R848-stimulation, which metacluster, and overlaid with the MSI of various cytokines. (F) Heatmap of all the pDC metaclusters showing the nMSI of various markers and cytokines/chemokines. Metaclusters derived from R848-stimulation are in blue box. (G) viSNE analysis of gated pDCs reveals 8 separate clusters that were gated on manually. Representative viSNE plot of patient 1 with cells from both media- and R848-stimulation overlaid with each cluster. (H) Heatmap of all the pDC viSNE clusters showing the nMSI of cytokines/chemokines, left. Percent pDCs within each cluster from each stimulation, right. (I) Representative manual gating of pDCs expressing IFNα vs. IL-8, vs. TNFα, vs. IL-6, and vs. IL-29.

We used mass cytometry to measure the ability of individual pDCs to produce multiple factors, a capacity known as “polyfunctionality”. To test for polyfunctionality, we used a CyTOF panel with antibodies against 10 cytokines/chemokines, IFNα, TNFα, IL-8, IFNλ1 (IL-29), IL-6, IL-1β, IL-10, CCL3, CCL4, and CXCL10. Polyfunctionality was initially explored by selecting pDCs, BDCA1 DCs, or BDCA3 DCs of each patient by manual gating and then further gating on each combination of cytokines/chemokines (Fig 7A and 7B). IFNα^+^ pDCs were highly polyfunctional: >90% produced two or more additional factors. Remarkably, 5% of the IFNα-producing pDCs made five or more additional cytokines/chemokines (Fig 7A). IFNα^-^ pDCs were less polyfunctional: 33% did not make any of the factors in our panel and most (58%) made only 2 or 3. Approximately 74% of the pDCs expressed TNFα and 73% expressed IL-8, more than the 26% that make the signature cytokine, IFNα (Fig 7D). BDCA1 DCs had a comparable level of polyfunctionality as pDCs, while BDCA3 DCs were mostly negative for the factors we analyzed (Fig 7B).

We used two additional analysis methods to characterize pDCs and to ensure that our findings were consistent regardless of which analytical method was applied. In the first approach, the pDCs of each patient were selected by manual gating and then Phenograph was used to identify subpopulations across all three patients (Fig 7C and Fig 7E, Fig 7F) [26]. Four metaclusters of R848-stimulated pDCs were identified (Fig 7F, blue box). Three (metaclusters 8, 17, and 7) had a high IFNα normalized mean signal intensity (nMSI) and also highly expressed TNFα and IL-8; they had variable expression of IL-6, CCL3, CCL4, and/or IFNλ1 (IL-29). In a final analysis, pDCs were first clustered using viSNE [27] followed by manually gating (Fig 7C–7G, and 7H). This process delineated eight clusters (Fig 7G). Similar to the metaclusters (Fig 7F), the viSNE clusters with high expression of IFNα (viSNE clusters 5, 6, 8) also had high levels of IL-8, TNFα, and they had variable expression of IL-6 and IL-29 (Fig 7H, left); nearly all the cells in cytokine-producing clusters came from cells stimulated by R848 (Fig 7H, right), consistent with manual gating in Fig 7I.

### Healthy liver pDCs from perfusates are just as polyfunctional

**Fig 8:**
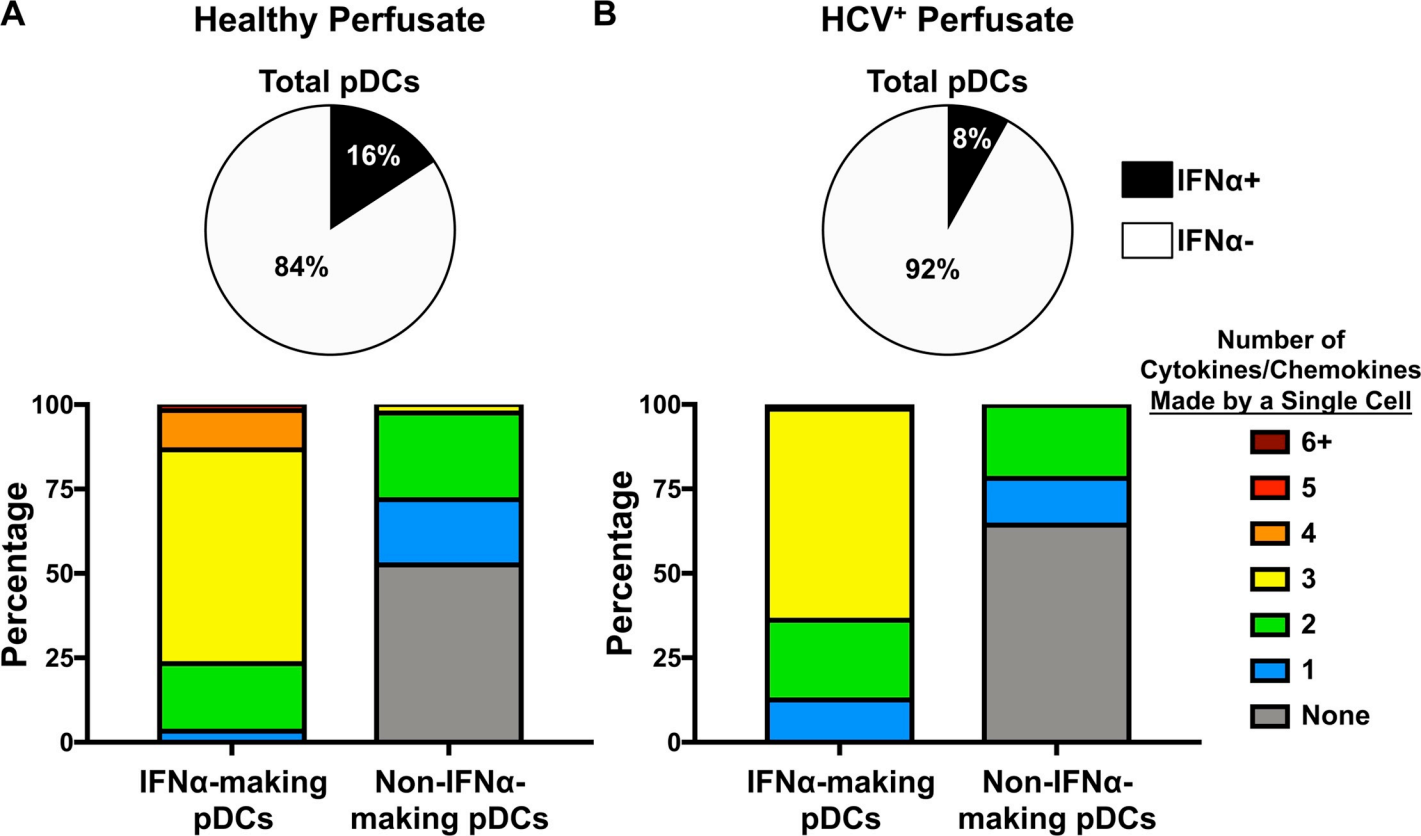
Healthy perfusate pDCs are equally polyfunctional. (A, top) Healthy perfusate mononuclear cells (PMCs) were assayed for IFNα+ pDCs after 4 hours stimulation with R848. (A, bottom) Polyfunctional profiling of IFNα-producing pDCs and non-IFNα-producing pDCs from the healthy perfusate. Each color represents the average number of cytokines/chemokines (of 10 in our panel) produced by a single pDC; IFNα-producing pDCs automatically start off producing 1 (or more) cytokine aka IFNα. (B, top) HCV-infected PMCs were assayed for IFNα+ pDCs after 4 hours stimulation with R848. (B, bottom) Polyfunctional profiling of IFNα-producing pDCs and non-IFNα-producing pDCs from the HCV-infected perfusate. Each color represents the average number of cytokines/chemokines (of 10 in our panel) produced by a single pDC; IFNα-producing pDCs automatically start off producing 1 (or more) cytokine aka IFNα.

To obtain cells from a liver of a patient with no underlying liver disease, we turned to the buffer solution that is used to transport donor livers. This “perfusate” is a validated source of liver immune cells and has been used in previous studies [28]. We obtained two perfusates, one from a healthy liver donor and the other from a HCV-infected liver donor. Both livers were deemed healthy enough for organ donation. We assayed the pDCs for polyfunctionality. After a four hour stimulation with R848, 16% of pDCs from the healthy perfusate made IFNα (Fig 8A, top), while only 8% of the HCV^+^ perfusate’s pDCs made IFNα (Fig 8B, top). We used the same CyTOF panel with antibodies against 10 cytokines/chemokines to explore polyfunctionality (Fig 8A and 8B, bottom). IFNα^+^ pDCs were more polyfunctional than their IFNα^-^ counterparts for both the healthy and HCV^+^ perfusates. Nonetheless, pDCs from a patient with no underlying liver disease were polyfunctional.

## Discussion

This is a detailed characterization of human liver pDCs from patients with a chronic hepatitis virus infection. It revealed that these rare innate immune cells are point sources of multiple immune activators and pro-inflammatory mediators, adding important new details about the fundamental capabilities of tissue-specific pDCs. Single cell mass cytometry (CyTOF) revealed that liver pDCs are the only IFNα producers among LMCs and revealed that only 15–40% synthesize it when stimulated with a TLR7/8 agonist (Figs 4D and 7D). Activated pDCs secreted large amounts of IFNα protein: ~2 million molecules per pDC per hour. While pDCs are traditionally known as “natural interferon producing cells,” our data revealed that they produce an array of bioactive molecules. Approximately 20% of pDCs make IFNα plus 4 of the other nine cytokines/chemokines we analyzed [TNFα, IL-8, IFNλ1 (IL-29), IL-6, IL-1β, IL-10, CCL3, CCL4, and CXCL10] and 13% make IFNα plus 5 or more (Fig 7A). Most IFNα^+^ pDCs expressed TNFα and IL-8, with variable amounts of IL-6, CCL3, CCL4, and IFNλ1 (IL-29). We did not determine the percentage of intrahepatic TNFα that is made by liver pDCs, but blood pDCs are major producers [29], suggesting that liver pDCs may be a significant source of this proinflammatory cytokine.

The perfusates from organ donors with livers healthy enough to transplant show that end-stage-liver disease is not a leading factor in whether or not pDCs are polyfunctional (Fig 8), nor does it seem to depend on HCV infection. The healthy perfusate pDCs were slightly more polyfunctional than the HCV-infected perfusate pDCs (Fig 8, bottom), suggesting that polyfunctionality is an innate characteristic of pDCs after stimulation through TLR7. It is worth noting that pDCs purified from liver tissue are more polyfunctional than the pDCs that did not pass through our lengthy extraction process (compare Fig 7A and Fig 8A, bottom). In future studies we hope to investigate pDCs from additional donors.

Four previous studies used flow cytometry to investigate pDC polyfunctionality; they demonstrated that individual pDCs can produce IFNα, TNFα, and IL-6 [30–33]. By using CyTOF, we were able to interrogate a larger number of factors than flow cytometry allows. We found that individual cells could express six or more cytokines and chemokines. It is likely that a broader CyTOF panel would reveal an even greater number of immune factors. One flow cytometric study demonstrated that gut pDCs of simian immunodeficiency virus-infected macaques secrete IFNα, TNFα, and MIP-1β [30]. Two showed that most activated human blood pDCs express two of the three factors analyzed [31, 32]. The fourth revealed that more than 95% of blood pDCs make two or fewer cytokines out of the four that were tested (IFNα, TNFα, IL-6, and IFNγ) after stimulation with R848 [33]. In addition, using a combination of single-cell RNA sequencing and single-cell cytokine analysis, Wimmers *et al*. recently reported that only a fraction of pDCs make IFNα, while most make TNFα, consistent with our data [25]. With our more comprehensive panel (10 cytokines and chemokines were analyzed) using CyTOF, we were able to show that liver pDCs, and similarly liver BDCA1 DCs, are highly polyfunctional for cytokine/chemokine production. Traditionally, polyfunctionality has been attributed to T cells and interpreted as an indicator of high functional capacity. Further studies are needed to understand the biological significance of having a single dendritic cell acting as a beacon of secreting multiple immune modulators. We postulate that polyfunctionality is important because it allows a single pDC to activate multiple signaling pathways on target cells, potentially raising the response to higher levels than could be achieved through the maximal activation of a single pathway. The exact composition of the cytokine/chemokine mix may also be important for eliciting appropriate responses.

Many recent studies provide novel information about the heterogeneity of pDCs. This heterogeneity may influence which pDCs acquire polyfunctionality. Alculumbre *et al*. demonstrated that when blood pDCs were stimulated with either influenza or CpG for 24 hours, the population matured into three distinct functional groups [34]. One subset produced IFNα, another stimulated T cells and a third had elements of both. At their 4 hour time point, the three subsets were not apparent. In addition, single cell RNA sequencing revealed that there are cells within the typical pDC gate that are not pDCs [20, 35]. Villani *et al*. showed that these pDC-like cells express *AXL* and *SIGLEC1/6* but in fact function like conventional DCs by activating T cells [35]. Michea *et al*. showed that the microenvironment can increase pDC heterogeneity [36]. MacParland *et al*. demonstrated that the liver microenvironment changes the phenotype of resident macrophages [37] suggesting that the liver microenvironment may impact the phenotype of liver pDCs.

Our study sheds new light on the paradoxical absence of detectable type I IFN mRNA in the HCV-infected liver despite the central role IFNα plays in host viral defenses. RT/qPCR data revealed that while IFNα/β mRNAs were not detectable in whole liver RNA extracts, confirming published findings [15, 16], they were readily detected in extracts of isolated liver leukocytes, demonstrating that purifying LMCs prior to mRNA analysis improved the signal-to-noise ratio in the RT/qPCR assay.

Importantly, type I IFN mRNAs were detected in whole liver using microarrays, indicating that mRNA expression occurred in the whole liver and did not require cell isolation. Consistent with this, the frequency of liver pDCs strongly correlated with expression of BT module of the IFNα response and three monocyte-specific modules, indicating that pDCs activate surrounding immune cells. The liver pDC frequency also strongly correlated with the percentage of HCV RNA in double-stranded form, which provides additional evidence that IFNαs were produced *in vivo*; published data establish that IFNα increases the percentage of double-stranded HCV RNA [12].

R848-stimulation strongly induced IFNα genes in LMCs *in vitro*, as demonstrated by RT/qPCR and transcriptomic analysis. The amount of IFNα produced in response to R848 strongly correlated with the frequency of liver pDCs and with expression of the “Immune activation–generic cluster” in whole liver, suggesting that pDCs activate multiple antimicrobial, inflammatory, and immune response pathways in liver immune cells, as depicted in S4 Fig. pDCs retain the ability to respond to TLR ligands even in the face of HCV.

Our study revealed interesting differences between pDCs in the liver and blood during chronic HCV infection. Liver pDCs were more highly activated, as indicated by higher expression of HLA-DR and *ex vivo* LMCs had higher expression of the “Immune Activation–Generic Cluster” and “TLR and Inflammatory Signaling” genes than *ex vivo* PBMCs (Fig 6E and S2B Fig). LMCs secreted more IFNα than PBMCs. Some of the observed differences may reflect the different procedures used to prepare PBMCs and LMCs, the latter were exposed to collagenase/DNase and mechanical disruption, which may have activated the liver pDCs. However, single cell RNA sequencing studies have shown that the microenvironment plays an important role in shaping the phenotype and function of immune cells [36, 37]. Importantly, LMCs remained responsive to TLR agonists during *ex vivo* culture, indicating that whatever effect the extraction process might have had it did not render the cells refectory to further activation. Additionally, despite on-going exposure to viral proteins like pathogen-associated molecular patterns (PAMPs) and cellular debris (danger-associated molecular patterns), pDCs remained functional.

When stimulated for four hours, LMCs secreted minimal IFNλ, whereas liver BDCA3^+^ DCs produced abundant IFNλ3 in response to 24 hours of stimulation [38] indicating that our experimental conditions were not optimal for IFNλ production. This suggests that a four hour time course is not appropriate for analyzing type III IFN responses. While acknowledging the importance of type III IFNs, we consider it likely that pDCs play an important role in liver immune responses during chronic HCV infection because the antiviral signature in LMCs was more strongly induced by the TLR-7/8 ligand than by the TLR-3 ligand. Because pDCs express TLR-7 and not TLR-3 [24], this finding suggests that pDCs, and by extension IFNα, stimulate antiviral defenses in the HCV-infected liver (S4 Fig). Our results are consistent with evidence that blood pDCs make IFNα in response to cell culture-derived HCV [10], a TLR-7 agonist.

In addition to LMCs, hepatocytes, sinusoidal endothelial cells, and other liver cells can produce IFNs in response to stress, including HCV infection. Hepatocytes secrete greater amounts of IFNλs than IFNαs [19]. Liver endothelial cells make primarily IFNλs after HCV exposure [39]. Type I and type III IFNs have distinctive effects. IFNα is more effective at inhibiting HCV replication *in vitro* [39], but IFNλ induces a more prolonged ISG induction [40]. Moreover, HCV infection of primary human hepatocytes causes a down-modulation of IFNAR1[41]. This down-modulation, if it occurs during chronic HCV infection, could protect HCV from antiviral defenses and foster chronic inflammation.

After successful HCV treatment, expression of some *IFNA* genes may increase [17]. If expression continues into the post-cure period, it could drive persistent liver inflammation, while also helping to suppress HCV recrudescence. Liver injury and inflammation continues in up to 66% of patients cured of HCV [42] and the immunopathology pre- and post-cure may involve some of the same molecular pathways. Pathologists were recently warned that the histopathology of liver transplant patients *cured* of HCV so closely resembles that of *chronic* infection that the conditions can be easily mistaken for each other [43]. Inflammation increases cancer risk; the HCC risk in cured cirrhotic patients remains elevated, up to 5% annually [44].

A limitation of the study is that most of the samples came from HCV-infected patients with end-stage liver disease and/or hepatocellular carcinoma. Future experiments need to be done on liver pDCs from additional sources.

In summary: Our study provides important new details about primary human liver pDCs and their activity during chronic HCV infection. The investigation used a novel combination of CyTOF, molecular techniques, cytokine quantitation, and cell purification methods and provided evidence that activated liver pDCs produce large quantities of IFNα. The liver pDC response to stimulation was heterogeneous, as also reported by Wimmers *et al*. for blood pDCs [25]. A minority of liver pDCs produced IFNα and most IFNα^+^ pDCs also expressed 2 or more of the other nine cytokines/chemokines we examined. Liver BDCA1^+^ DCs were also highly polyfunctional. The circuits regulating gene expression in polyfunctional liver DC subsets merit investigation as the orchestrators of complex immune responses and as potential therapeutic targets.

## Methods

### Ethics statement

This is a prospective study of specimens and medical records of 19 HCV-positive adults who received a liver transplant at the Mount Sinai Medical Center between 11/2013 and 8/2014 and who gave written informed consent. Blood for research and clinical testing was collected before surgery. Explants of three additional anonymous HCV-infected patients were also analyzed. Perfusates of two anonymous liver donors, one healthy and one HCV-infected, were collected and analyzed. The study was approved by Mount Sinai’s IRB in accordance with Helsinki guidelines. No explants were obtained from prisoners or other institutionalized persons.

### Preparation of LMCs and PBMCs

Specimens were brought to the laboratory at a median of 45 min post-explantation. The liver capsule was removed. Tissue was minced, washed in Hank’s balanced salt solution (HBSS)/1% fetal calf serum (FCS), incubated in RPMI/5% FCS/0.1 mg/mL collagenase/50 μg/mL DNase at 37°C for 30 min, shaking every 5 min. Tissue was pressed through stainless steel mesh while washing with HBSS/1% FCS. Cells were washed and resuspended in HBSS/1% FCS and filtered through 100μm nylon mesh. Percoll gradients were used to purify LMCs from the filtrates [45, 46] and from PBMCs.

### Preparation of perfusate immune cells

Perfusates were kept on ice during transportation and brought to the laboratory after anhepatic phase of liver transplantation was complete. Perfusates were spun down and resuspended in HBSS/1% FCS. Percoll gradients were used to purify PMCs from the perfusates.

### Flow cytometry

The flow cytometry panel for DC subsets appears in S3 Table. Cells were stained with the surface stain panel, then fixed with 2% paraformaldehyde solution (Thermo Scientific) in PBS. Samples were run on an LSR Fortessa (BD) and analyzed using Flojo.

### Cell stimulation

Without BFA: One million PBMCs or LMCs per 0.5mL media were incubated in RPMI with 10% FBS for four hours alone or with 1 μg/mL R848 or with 50 μg/mL Poly I:C at 37°C. Supernatants were collected for proteomic analysis. Cells were collected in Trizol (Life Technologies) for RT/qPCR and microarray analysis. With 1:1000 BFA (eBioscience): Up to ten million PBMCs, LMCs, or PMCs in 0.5mL media were incubated in RPMI with 10% FBS for four hours alone or with 1 μg/mL R848 at 37°C. Cells were collected for CyTOF antibody staining and acquisition.

### RNA extraction and RT/qPCR

RNAs were purified as before [47]. cDNA was made using SuperScriptIII First-Strand Synthesis (Invitrogen) and amplicons were quantified using the LightCycler480 SYBR Green II Master kit (Roche). Expression of genes was calculated using the ΔΔC_t_ method normalized to *RPS11* and to expression of the PBMC *ex vivo* sample. Primers for *IFNA1*, *IFNB*, *IFNL1*, *IFNl2/3*, *RPS11*, and *TNFA* were described previously [48]. Double stranded HCV RNA was quantified as described previously [47].

### Proteomics

Luminex multiplex cytokine assays (Millipore) quantified IFNα2a/b, IFNλ1 (IL-29), IFNλ2 (IL-28A), IFNλ3 (IL-28B), interferon gamma-induced protein 10 (IP10 aka CXCL10), interleukin 6 (IL-6), IL-10, and TNFα.

### Microarrays

Profiling data from Illumina Human-HT-Expression Beadchips were normalized using GenomeStudio’s quantile method. GenePattern was used for gene set enrichment analysis (GSEA) and single sample GSEA (ssGSEA) of immune pathways [49] using BT modules [50] and KEGG pathways. A false discovery rate (FDR) below 0.25 was considered statistically significant. LMCs/PBMCs: To obtain sufficient RNA, LMC samples of matched pairs of patients were pooled. Matching was based on age, gender, HCV genotype, baseline HCV RNA, natural MELD score, and HCC (yes/no). PBMCs were pooled similarly. Whole liver: Whole liver microarray data of 11 of the 19 patients consented for this study was used as before [47].

### CyTOF

Panel presented in S5 Table. Samples were washed, fixed, and permeabilized (eBiosciences) then stained with intracellular antibodies. Samples were stored at 4°C in Ir intercalator (Fluidigm) in 2% formaldehyde until acquisition. Before acquisition, samples were mixed with EQ4 Element Beads (Fluidigm) and were acquired on a CyTOF2 (Fluidigm). Data were normalized using bead-based normalization in the CyTOF software and gated to exclude beads, dead cells, and doublets. Method 1: Gated pDCs were analyzed using an automated CyTOF data analysis pipeline at the Mt. Sinai HIMC, which uses an R-based implementation of Phenograph [26], an agonistic clustering method that utilizes the graph-based Louvain algorithm for community detection and identifies a hierarchical structure of distinct phenotypic communities. We utilized dynamic activation markers and intracellular cytokines as clustering parameters to resolve functional heterogeneity within the pDC population. Phenotypic clusters from 3 donors were meta-clustered identify consistent populations that could be reproducibly detected across individuals, thereby generating a consistent cluster structure across all samples in the dataset, while preserving the diversity and heterogeneity of all subpopulations. Method 2: viSNE was used to cluster the single-cell pDC data, creating t-distributed stochastic neighbor embedding (tSNE) plots in Cytobank [27]. viSNE uses a dimensionality-reducing algorithm to express multi-dimensional data in two dimensions. Canonical cell surface markers were then analyzed to identify cell populations overlaid on the viSNE map or manually identified clusters were gated on and overlaid on the viSNE map.

### IFNα production per pDC

The Luminex-measured mean quantity of secreted IFNα was divided by the incubation time to determine production per hour. This quantity was divided by the molecular mass of IFNα and multiplied by Avogadro’s constant. The result (the molecules of IFNα secreted per hour) was divided by the mean number of IFNα-producing pDCs per reaction, which was determined by multiplying the number of LMCs per reaction by the frequency of IFNα-producing pDCs as determined by CyTOF.

### Statistical analysis

GraphPad Prism was used. Paired and unpaired t-tests were performed. Pearson’s correlation coefficient was used for correlations.

## Supporting information

S1 Fig Identification of 3 intrahepatic dendritic cell populations by multiparameter flow cytometry. Intrahepatic mononuclear cells from a representative liver were stained with a nine-color antibody panel: CD45, CD3, CD19, CD20, HLA-DR, CD14, CD16, CD123, BDCA1, BDCA3, and CD56. Innate immune mononuclear cells were selected based on viability (live/dead), unicellularity (singlets/other), CD45 expression (CD45^+^/CD45^-^), intracellular complexity (non-granulocytes/granulocytes), and lack of expression of lineage markers (CD3^-^, CD19^-^, CD20^-^/CD3^+^, CD19^+^, CD20^+^).
(TIF)

S2 Fig Liver mononuclear cells have greater immune activation both *ex vivo* and after stimulation than PBMCs. GSEA of gene sets from the blood transcriptome (BT) modules related to: (A) “Antiviral IFN signature” comparing R848-stimulated LMCs and *ex vivo* LMCs; (B) “TLR and inflammatory signaling” comparing *ex vivo* LMCs and R848-stimulated LMCs; (C) “Antiviral IFN signature” comparing *ex vivo* LMCs and PolyI:C-stimulated LMCs; and (D) “Antiviral IFN signaling” comparing LMCs and PolyI:C-stimulated LMCs. Pathways with a false discovery rate (FDR) below 0.25 were considered significant. Genes contributing to pathway enrichment (leading edge genes) are boxed and in heat maps below.
(TIF)

S3 Fig Secretion of pro- and anti-inflammatory cytokines and chemokines. LMCs and matched PBMCs were stimulated with R848, PolyI:C or media alone for cytokine production. Total secretion (pg/mL) of (A) TNFα, (B) CXCL10 (IP10), (C) IL-10, and (D) IL-6. Horizontal bars depict the mean ± SD. N = 17, paired t-tests.
(TIF)

S4 Fig Model of liver pDCs’ role in inflammation and ISG induction. (TIF)

S1 Table Patient characteristics at transplant. (DOCX)

S2 Table Flow cytometry panel gating strategy. (DOCX)

S3 Table Flow cytometry panel. (DOCX)

S4 Table Blood transcription module pathways. (DOCX)

S5 Table CyTOF panel. (DOCX)
