# Supplementary figures and images for: Individual liver plasmacytoid dendritic cells are capable of producing IFNα and multiple additional cytokines during chronic HCV infection
Source: PLoS Pathog. 2019 Jul 29;15(7):e1007935. doi: 10.1371/journal.ppat.1007935 (PMC6687199; doi:10.1371/journal.ppat.1007935)

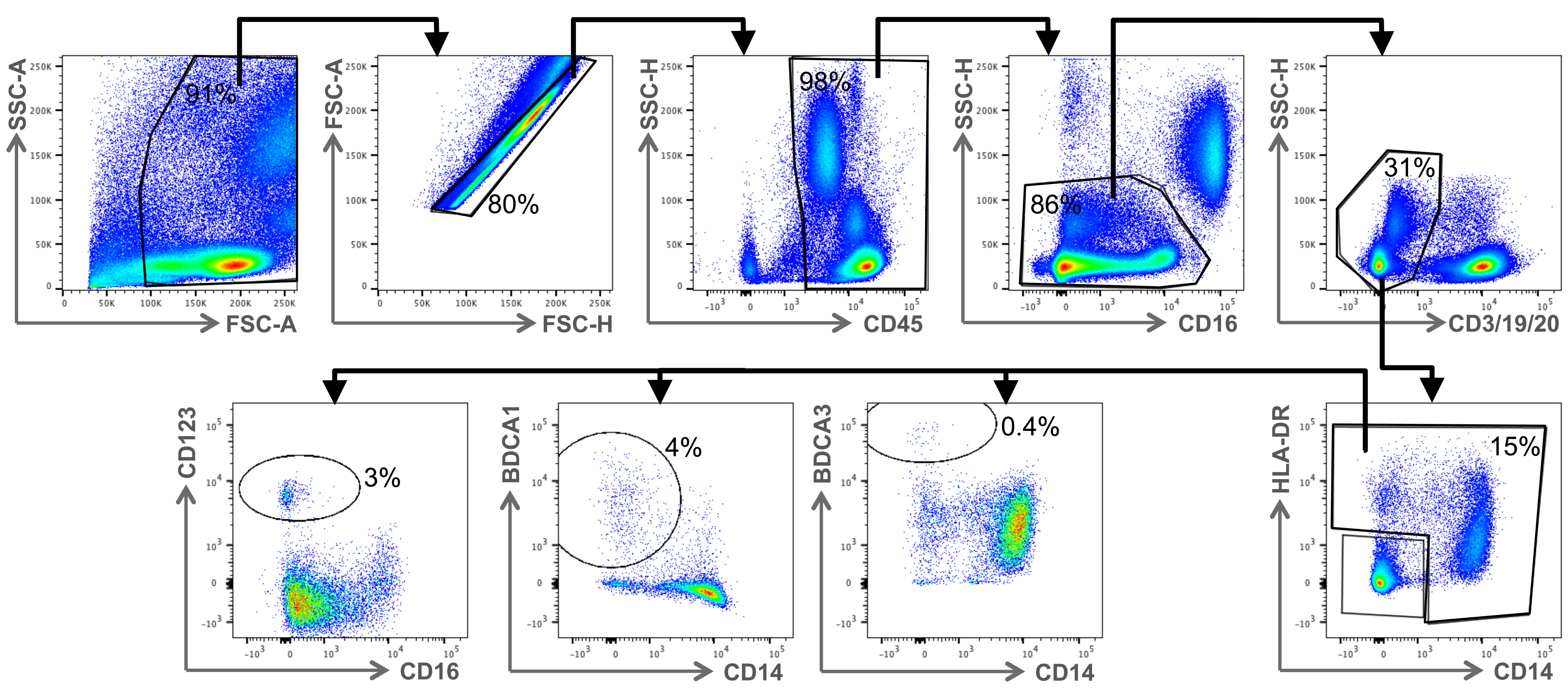

Supplement: S1 Fig — Intrahepatic mononuclear cells from a representative liver were stained with a nine-color antibody panel: CD45, CD3, CD19, CD20, HLA-DR, CD14, CD16, CD123, BDCA1, BDCA3, and CD56. Innate immune mononuclear cells were selected based on viability (live/dead), unicellularity (singlets/other), CD45 expression (CD45+/CD45-), intracellular complexity (non-granulocytes/granulocytes), and lack of expression of lineage markers (CD3-, CD19-, CD20-/CD3+, CD19+, CD20+). (TIF) [file ppat.1007935.s001.tif]

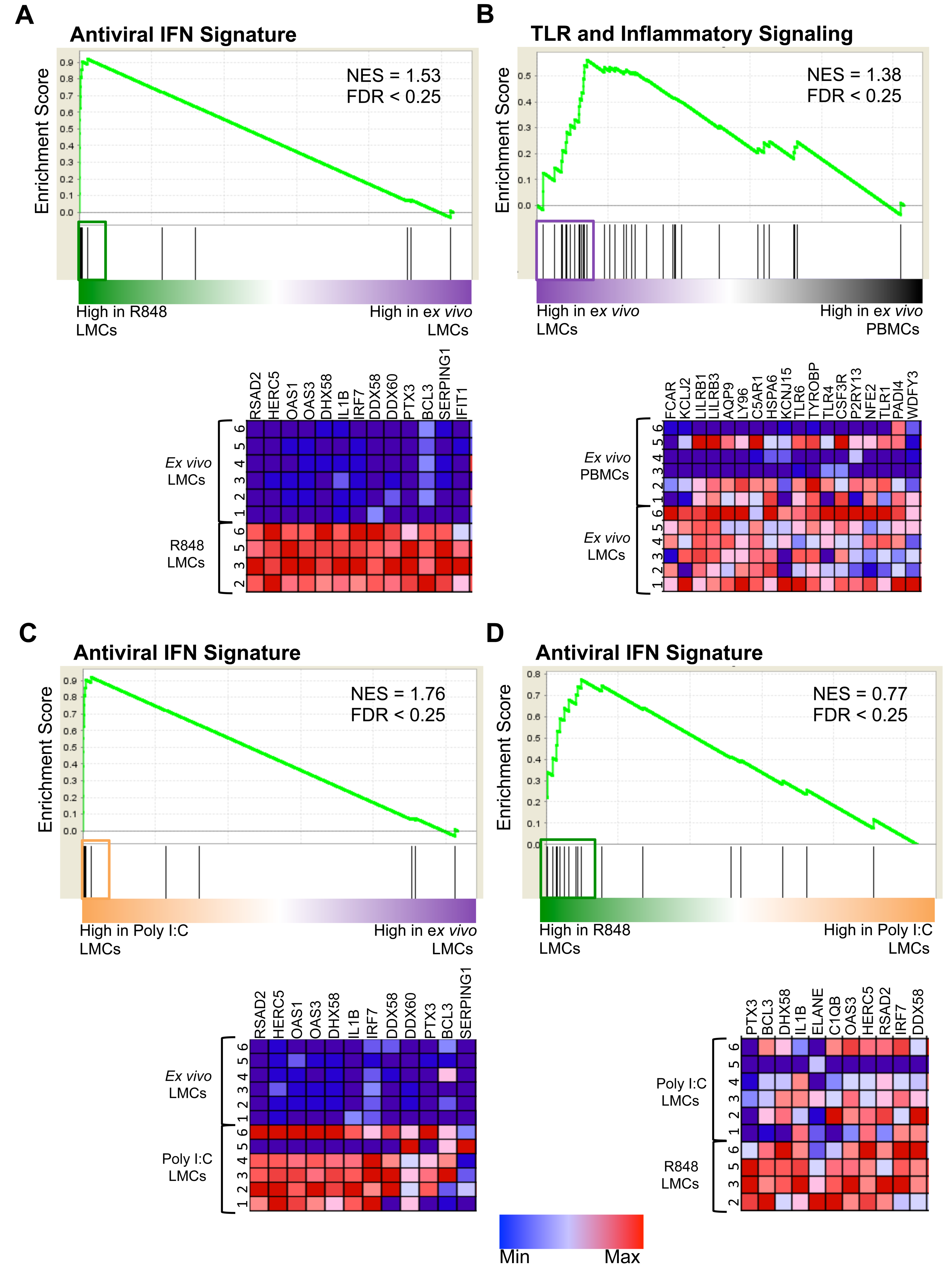

Supplement: S2 Fig — GSEA of gene sets from the blood transcriptome (BT) modules related to: (A) “Antiviral IFN signature” comparing R848-stimulated LMCs and ex vivo LMCs; (B) “TLR and inflammatory signaling” comparing ex vivo LMCs and R848-stimulated LMCs; (C) “Antiviral IFN signature” comparing ex vivo LMCs and PolyI:C-stimulated LMCs; and (D) “Antiviral IFN signaling” comparing LMCs and PolyI:C-stimulated LMCs. Pathways with a false discovery rate (FDR) below 0.25 were considered significant. Genes contributing to pathway enrichment (leading edge genes) are boxed and in heat maps below. (TIF) [file ppat.1007935.s002.tif]

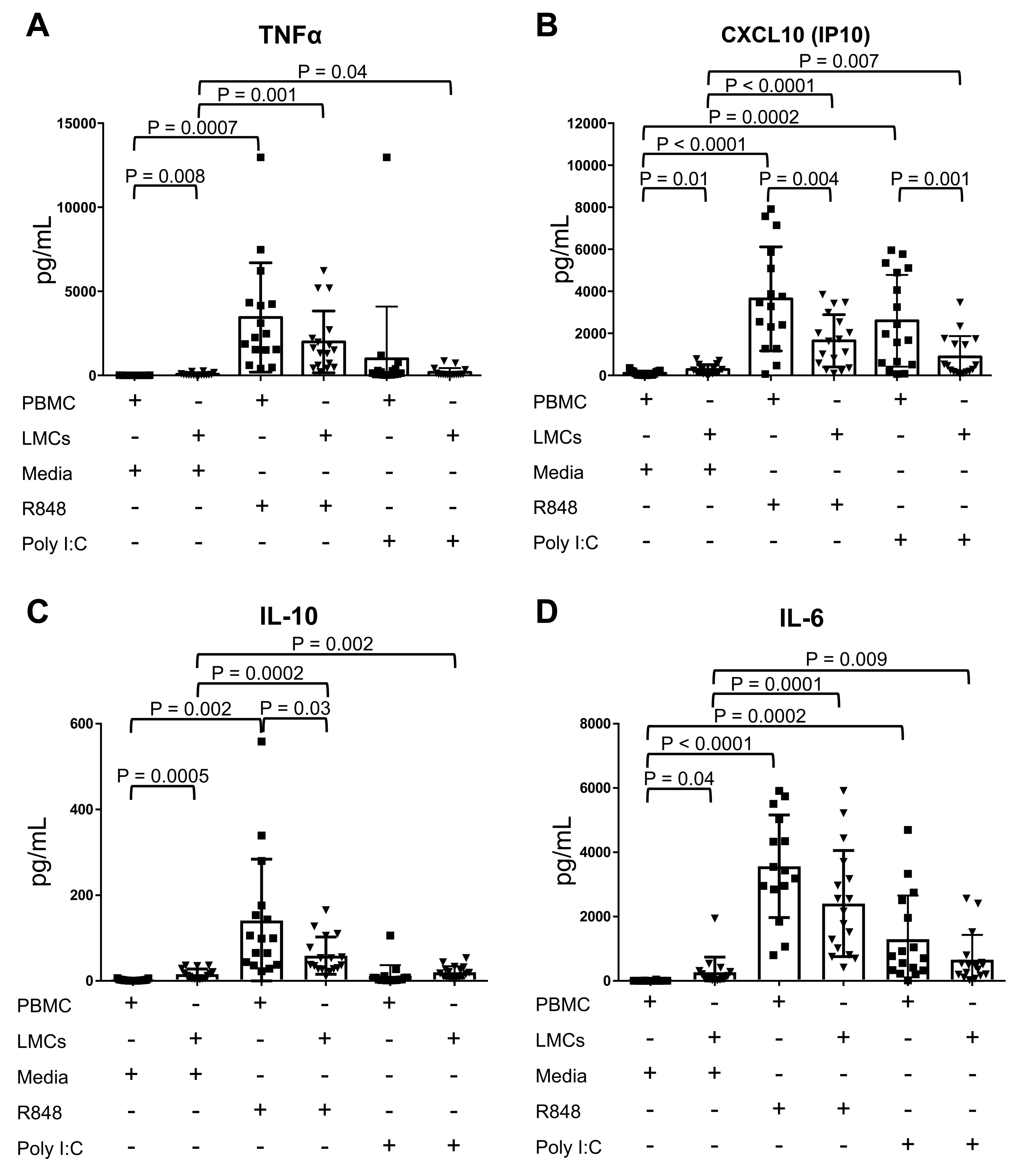

Supplement: S3 Fig — LMCs and matched PBMCs were stimulated with R848, PolyI:C or media alone for cytokine production. Total secretion (pg/mL) of (A) TNFα, (B) CXCL10 (IP10), (C) IL-10, and (D) IL-6. Horizontal bars depict the mean ± SD. N = 17, paired t-tests. (TIF) [file ppat.1007935.s003.tif]

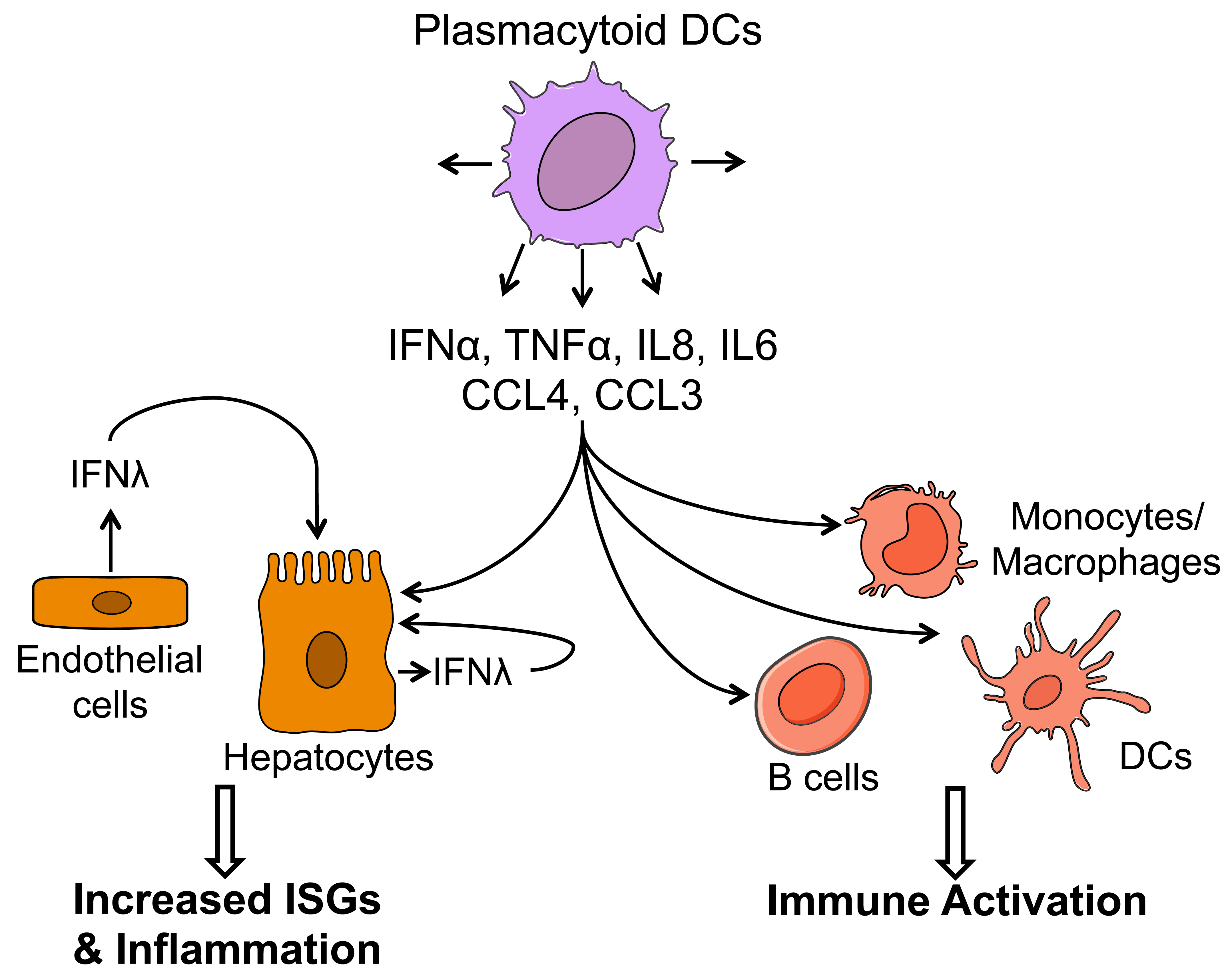

Supplement: S4 Fig — (TIF) [file ppat.1007935.s004.tif]
