# Supplementary material for: Individual liver plasmacytoid dendritic cells are capable of producing IFNα and multiple additional cytokines during chronic HCV infection
Source: PLoS Pathog. 2019 Jul 29;15(7):e1007935. doi: 10.1371/journal.ppat.1007935 (PMC6687199; doi:10.1371/journal.ppat.1007935)
Supplement: S1 Table — (DOCX) [file ppat.1007935.s005.docx]

| Supplementary Table 1. Patient Characteristics at Transplant. | |
| --- | --- |
| Clinical Characteristics  Median (IQR), N (%) | **Patients**  N = 19 |
| Age (years) | 62 (59 – 65) |
| Male | 15 (79%) |
| Race  Asian  Black  Caucasian  Mixed  Other | 1 (5%)  3 (16%)  13 (68%)  1 (5%)  1 (5%) |
| Hispanic | 4 (21%) |
| Reason for Transplant  Hepatocellular carcinoma  End-stage liver disease | 13 (68%)  6 (32%) |
| HCV Genotype  1  2, 3, or 4 | 13 (68%)  6 (32%) |
| Serum HCV RNA (IU/mL) | 8.7x10^4^ (1.4x10^4^ - 2.2x10^5^) |
| Liver HCV RNA (RNA copies per 100 ng total RNA)  Total HCV RNA  Single-stranded HCV RNA  Double-stranded HCV RNA  Percentage of Liver HCV RNA in double-stranded form | 2.5x10^6^ (2.8x10^3^ – 3.4x10^6^)  9.7x10^2^ (1.2 – 1.6x10^6^)  5.4x10^5^ (2.0x10^3^ – 1.5x10^6^)  51% (33 – 74%) |
| *IL28B* Genotype  Declined consent  CC  CT  TT | 9 (47%)  1 (5%)  5 (26%)  4 (21%) |
| Platelets (x10^3^/μL) | 75 (57 – 95) |
| INR | 2 (1 – 2) |
| ALT (U/L) | 36 (27 – 69) |
| AST (U/L) | 62 (38 – 103) |
| Natural MELD | 18 (13 – 32) |
| IQR, interquartile range; INR, international normalized ratio; ALT, alanine aminotransferase; AST, aspartate aminotransferase; MELD, model for end-stage liver disease | |
