# Supplementary material for: Individual liver plasmacytoid dendritic cells are capable of producing IFNα and multiple additional cytokines during chronic HCV infection
Source: PLoS Pathog. 2019 Jul 29;15(7):e1007935. doi: 10.1371/journal.ppat.1007935 (PMC6687199; doi:10.1371/journal.ppat.1007935)
Supplement: S2 Table — (DOCX) [file ppat.1007935.s006.docx]

| Supplementary Table 2. Flow Cytometry Panel Gating Strategy. | |
| --- | --- |
| Population | **Gating** |
| Plasmacytoid Dendritic Cells | Live Singlet CD45+ Granulocyte- CD3- CD19- CD20- CD16- CD123+ |
| BDCA1 Dendritic Cells | Live Singlet CD45+ Granulocyte- CD3- CD19- CD20- CD14- BDCA1+ |
| BDCA3 Dendritic Cells | Live Singlet CD45+ Granulocyte- CD3- CD19- CD20- CD14- BDCA3+ |
