# Supplementary material for: Individual liver plasmacytoid dendritic cells are capable of producing IFNα and multiple additional cytokines during chronic HCV infection
Source: PLoS Pathog. 2019 Jul 29;15(7):e1007935. doi: 10.1371/journal.ppat.1007935 (PMC6687199; doi:10.1371/journal.ppat.1007935)
Supplement: S3 Table — (DOCX) [file ppat.1007935.s007.docx]

| Supplementary Table 3. Flow Cytometry Panel. | | |
| --- | --- | --- |
| Antibody & Conjugate | **Vendor/Clone** | **Catalog Number** |
| Anti-human CD45 V500 | BD/HI30 | 560777 |
| Anti-human CD3 FITC | Biolegend/UCHT1 | 300406 |
| Anti-human CD19 FITC | Biolegend/HIB19 | 302206 |
| Anti-human CD20 FITC | Biolegend/2H7 | 302304 |
| Anti-human HLA-DR AF700 | Biolegend/LN3 | 327014 |
| Anti-human CD14 APC Cy7 | Biolegend/HCD14 | 325620 |
| Anti-human CD16 BV650 | Biolegend/3G8 | 302041 |
| Anti-human CD123 PE Cy7 | BD/7G3 | 560826 |
| Anti-human CD1c AF647 | Biolegend/L161 | 331510 |
| Anti-human CD141 PE | BD/1A4 | 559781 |
| Anti-human CD56 PerCP Cy5.5 | Biolegend/HCD56 | 318322 |
