# Supplementary material for: Individual liver plasmacytoid dendritic cells are capable of producing IFNα and multiple additional cytokines during chronic HCV infection
Source: PLoS Pathog. 2019 Jul 29;15(7):e1007935. doi: 10.1371/journal.ppat.1007935 (PMC6687199; doi:10.1371/journal.ppat.1007935)
Supplement: S4 Table — (DOCX) [file ppat.1007935.s008.docx]

| Supplementary Table 4. Blood Transcription Module Pathways. | | |
| --- | --- | --- |
| Pathway* | **Corresponding Figure** | **Genes** |
| Antiviral IFN signature | Sup. Fig. 2A, 2C & 2D | *IFIH1, ELANE, SERPING1, IL1B, RSAD2, IFIT1, RARA, DDX58, FCER1A, DHX58, PTX3, CARD9, OAS1, OAS3, PML, ANXA3, HERC5, DDX60, CXCL10, IRF7, C1QB, BCL3* |
| BCR signaling | Fig. 3F | *INPP5D, SYK, PIK3CD, VAV1, PLCG2, PIK3R5, PIK3R3, RAC2, LYN, PTPN6, BTK, BLNK* |
| Cell division stimulated CD4+ T cells | Fig. 3F, 3H | *BRCA1, GINS2, CENPM, BRCA2, CDKN3, ANLN, CEP55, HMMR, POLE2, CDCA2, GINS1, NCAPG2, CDCA7, FANCD2, UBE2T, DEPDC1B, FANCL, FAM72A, FAM72B, FAM72C, FAM72D, FANCI, DSCC1* |
| Cell movement, adhesion & platelet activation | Fig. 3C, 3E | *PDGFA, ITGB3, PTK2, TNS1, SELP, MPP1, PROS1, KIF26A, TPM1, GAB1, NAV1, HBEGF, CTGF, COL5A1, ALOX12, ESAM, GP6, CALD1, CD9, SEMA6A* |
| Chaperonin mediated protein folding (I) | Fig. 2C | *CCT8, ATIC, CCT2, CCT3, NOP56, CCT7, CCT4, CCT5, HSPD1, SUCLG1, HSPE1, SSBP1, FBXW2, CCT6A* |
| Cytoskeletal remodeling (enriched for SRF targets) | Fig. 3C | *ACTN1, PPAP2B, VCL, MYLK, TPM1, NR2F2, CTGF, THBS1, CALD1, TPM2* |
| Enriched in activated dendritic cells/monocytes | Fig. 2C | *CXCL1, DRAM1, G0S2, SOD2, SIRPA, IL1RN, NINJ1, IL13RA1, TNFAIP2, SLC31A2, BASP1, MARCKS, FAM129A, ANPEP, SLC15A3, IL1B, IL8* |
| Enriched in antigen presentation (II) | Fig. 2C | *CD53, IL10RA, ITGA4, FYB, HLA-DRA, HLA-DRB1, HLA-DRB4, HLA-DRB5, HLA-DRB3, HCLS1, PTPRC, HLA-DPB1, ITGB2, GIMAP6, EVI2B, PLEK, SLA, SELL, IRF8, FGL2, ITGAL, ITGAM, AIF1, HLA-DMA* |
| Enriched in antigen presentation (III) | Fig. 2C | *FYB, CD53, HLA-DRA, ITGB2, ICAM1, PLEK, HLA-DRB1, LAPTM5, HLA-DRB4, HLA-DRB5, PTPRC, HLA-DRB3, EVI2B, HLA-DMB, AIF1, HLA-DMA* |
| Enriched in B cells (IV) | Fig. 3F | *FAIM3, LOC284749, CPNE5, TPD52, CIITA, IGHA1, TLR10, IGHG1, CELSR1, FAM30A, CDK14, PAWR, CD180, MEF2C, BTK, HLA-DOA* |
| Enriched in B cells (VI) | Fig. 5H | *CR2, PCDH9, TSPAN13, HLA-DOB, CD72, PTPRK, CD22, P2RX5, BTLA, MS4A1, PPAPDC1B, CXCR5, ABCB4, CD200, FCRL1, FCRL2, CD79A, CD79B, ADAM28, CD19* |
| Enriched in monocytes (surface) | Fig. 2C | *LTBR, CD4, TNFRSF1B, MCTP1, S1PR3, EMR1, C19orf59, SLC24A4, STEAP4, C1orf162, SLC16A3, NFAM1, TMEM176B, HLA-DMB, IL1R2, AGPAT9, TNFSF13B* |
| Formyl peptide receptor mediated neutrophil response | Fig. 3F, 3G | *NCF2, SLC11A1, CAMK1, FPR1, IGSF6, SECTM1, FCGR2A, PILRA, NCF1C, PAK1* |
| Hox cluster III | Fig. 3C, 3D | *HOXB3, HOXB6, HOXB7, HOXA11, HOXA10, HOXA7, MEIS1, HOXA5, HOXA4, HOXB4, MEIS2, HOXA9* |
| Hox cluster IV | Fig. 3C | *HOXB9, HOXB2, HOXB6, HOXB7, HOXB4, HOXB5, HOXA5, HOXA4, HOXA11, HOXA9* |
| Immune activation – generic cluster | Fig. 5F, 6E | *ELANE, SAMD13, CAMK4, GPR109B, BANK1, DYSF, MACROD2, MS4A4A, PRRG4, ORM1, C19orf59, ERI2, LAT, PPIL4, CSRP2, MAL, CD1B, RASGRP4, MGST1, PRG2, VPREB1, VPREB3, ENG, GRAP2, NCAPD2, NCAPD3, BLM, TCN1, SLC22A4, ANPEP, SIRPB1, CD19, RETN, MBOAT2, CLEC5A, PLA2G7, CIITA, PTX3, SMC4, P2RY13, S100A8, RNF150, KIAA0513, C10orf10, MANSC1, LTF, PRAM1, SLC27A2, ACPP, F5, HMGB2, CHAF1A, CHAF1B, HLA-DRA, SULF2, CDK14, CXCR2, KLF5, DEFA4, DEFA1, JAG1, FPR2, SLC24A4, CTSG, TCL1A, C3AR1, NUP37, CA4, OGN, CMTM2, LILRA5, LILRA3, LIN7A, HNRPLL, DKFZp451A211, CHI3L2, CHI3L1, CYP4F2, CYP4F3, B3GNT5, ORC6L, ROPN1L, CRISPLD2, CENPL, CRISP3, BARD1, FAM69B, CCDC150, RBBP8, LY96, ACCN2, GH1, AZU1, DBF4, CD300C, LOC90925, EMR1, VANGL1, ARHGEF10L, OSCAR, MGAM, CCNE1, CEACAM1, CEACAM6, TACSTD2, BMX, PLXDC2, G0S2, JAM3, VNN2, VNN1, IGFBP7, BLVRB, GALNT6, STK32B, C15orf23, MEF2C, DTYMK, GPR97, BTK, CD72, CXorf50B, RFC2, PRKAR2B, TREM1, MCTP1, GAS2L1, NLRP3, ADAP2, RANBP1, FGD4, ACPL2, LOC81691, CLC, GXYLT2, UNG, GAB1, TM6SF1, FAM43A, LOC284757, SLC22A15, BASP1, PTK2, SVIL, LCN2, BPI, MSH2, MEGF9, F13A1, VSTM1, IQGAP3, ARAP3, RAD54B, C13orf34, RGL1, CAMP, RGL4, LOC100130458, ST3GAL6, FCGR2C, MGC5566, PADI2, EBF1, DHCR24, CLEC4D, PFKFB4, ZNF467, TUBG1, MOSC1, MMP8, MMP9, SAC3D1, FKBP14, PTGS2, AMICA1, LOC643332, CLEC14A, TXNDC3, FAM46A, PSD3, INHBA, PRTN3, FCAR, BRCA2, HAL, LRRC4, CREB5, AQP9, TMEM176A, TMEM176B, CTSS, CXCL1, PSRC1, SMPDL3A, ADCY9, RUNX2, BST1, PID1, CD3E, CD3G, CSF2RB, DOCK5, LARGE, TSHR, ICOSLG, QPCT, JUP, NFE2, PAX5, CORO2A, CR1, PROK2, PEX5L, PRTFDC1, LOC100289612, SH2D1A, CD177, CD7, CD2, OLFM4, CCR9, CD9, SLC8A1, GPR84, KIF16B, ABCA13, LGALS3, BCL11B, RAD51C, SLPI, LRP1, CDA, SGK1, STX11, HLA-DOA, AGPAT9, CYFIP1, CDKN2C, FCGR3A, S100P, FCGR3B, FOXO1, NLRC4, ALDH1A1, MLLT4, CHIT1, SLCO4C1, LOC728606, LPCAT2, HK3, ITGAM, ANXA3, ANXA5, NRGN, MCM7, MCM3, IL18RAP, UPP1, SAMHD1, CEBP, OLR1, IMPA2, KCNK5 CKAP2, BCL6, KCNE3, LAT2, PKMYT1, FBP1, MS4A7, MS4A3, GSN, VSIG10, NDST3, LYZ, NAV1, LYN, MYB, PYGL, RNASE3, RNASE2, ENTPD1, RNASE6, CD22, TSPAN2, RBP7, CACNA2D3, ACSL1, TLR1, TLR7, ATP8B4, SLC19A2, GGTA1, CLEC12A, HP, GPBAR1, IL1R2, FLJ10357, BEX1, LRRK2, SCD, PCBP3, SERPINB10, TLR5, RHOU, SLC26A8, ARG1, TNFAIP6, ELOVL4, FAM105A, FAM150B, NCF4, GAS2L3, GNAI1, LGALS12, SLA2, C11orf75, DUSP6, FAM49A, EMILIN2, CTGF, CSE1L, STEAP4, MYCL1, TFF3, APOB48R, UBASH3A, RAB32, ESCO2, TRAT1, DOK3, IL1R1, NACC2, LRP12, PDGFC, KCNJ15, DACH1, LRG1, ACTN1, SLC40A1, SIGLEC9, SIGLEC5, FOLR3, ZNF608, MYO7B* |
| Integrin cell surface interactions (I) | Fig. 3C | *ITGA9, PDGFRA, PTK2, TNC, PDGFRB, EGFR, ITGA5, LAMB1, COL3A1, LAMB2, CAV2, MYL9, ITGA2B, KDR, ITGB3, COL5A2, COL5A1, COL4A2, COL4A1, LAMC1, FN1, CCND2, ITGAV, MET, COL1A2, COL1A1, COL6A1, COL6A3, COL6A2* |
| Interferons | Fig. 6F | *IFNA1, IFNA10, IFNA13, IFNA14, IFNA16 IFNA17, IFNA2, IFNA21, IFNA4, IFNA5, IFNA6, IFNA7, IFNA8, IFNB1, IFNE1 IFNK IFNW1, IL28A, IL28B, IL29* |
| Interferon alpha response (I) | Fig. 2C, 2D | *LHCGR, COL8A1, IMPG2, ITGB4, MMP12, TNR, IFNA7, IFNA4, SFN, LAMC2, ST14, ADAMTS20, FGF5, IFNA10, IFNA16, IFNA14* |
| Monocyte surface signature | Fig. 2C, 2E | *FXYD6, KCNE3, AMICA1, CD33, CD36, C5AR1, FCGR1B, APCDD1, LTBR, PECAM1, CYBRD1, GPR109B, PLXND1, FCAR, DYSF, FPR1, FPR2, HLA-DRB4, CSF3R, CCR1, C19orf59, TLR2, CD163, TLR1, TLR4, TLR5, TREM1, TNFSF12, CLEC12B, CLEC12A, EMR1, EMR2, LPPR2, IL1R2, MARCO, LILRB3, LILRB2, LILRB1, SLC11A1, MGAM C10orf54, TMEM71, SLC46A2, VNN2, KCNQ1, FCGR2A, TNFRSF1B, MBOAT7, CD93, ITGAM, SIRPB1, GPER, CD4, TNFRSF10B CCR2 CD302, PTAFR, MCTP1, FCER1A, S1PR3, P2RY13, SLC7A7, AGTRAP, CECR6, IL6R, ASGR1, ASGR2, TMEM154, LRP1, LRRC33, C1orf162, TMTC2, ABCA1, STEAP4, SPNS1, P2RX1, VSTM1, BRI3, NFAM1, TMEM55A, PYCARD, FCGRT, GLIPR1, MFSD1, SLC24A4, PTGIR, MS4A6A, P2RY2, SLC40A1, SLC16A3, ANO10, PTPRE, SIGLEC9, GPR133* |
| Platelet activation (III) | Fig. 3F | *TGFB1, EHMT2, TLN1, ACTN4, PKN1, SIPA1, ACAP1, GNB2, AP2M1, AKT1, MAP7D1, MLF2, ZYX, NBEAL2, DNM2, FLNA, HGS, PFN1, GNAI2, MAP2K2, PNPLA6* |
| Spliceosome | Fig. 2C | *SNRPE, SNRPD3, SNRPD1, LSM3, LSM5 SNRPG, RBMX, SF3B14, SNRPA, SNRPF, MAGOH, SNRPD2* |
| TLR and Inflammatory signaling | Sup. Fig. 2B | *FCGR1A, FCGR1C, HSPA6, NCF4, TLR6, C5AR1, FCGR1B, APOB48R, LILRB3, KCNJ15, LILRB1, PILRA, TYROBP, MGAM, FGR, P2RY13, CXCR2, NFE2, IRAK3, MYD88, FPR2, SIGLEC9, FCAR, DYSF, NPL, FPR1, ALOX5, FCGR2C, AQP9, FES, CSF3R, TLR7, LY96, LILRB2, ITGAX, KCNJ2, WDFY3, TLR2, TLR1, BST1, ANPEP, TLR4, TLR5, LILRA6, TLR8, PADI4* |
| Transmembrane transport (I) | Fig. 2C | *VDR, SLC7A7, CD36, SLC31A2, SLC4A1, TLR2, NLRP3, SLC11A1, ABCB6, CDH1, DAPK1, SLC14A1, AQP1, HK3, ATP1B2, AQP9, SLC25A21, SLC15A3, ABCG2, KCNH2, HMOX1, SLC22A4, RHAG, TLR4* |
| *Pathways are from Li *et al*. (2014) Nature Immunology | | |
