# Supplementary material for: Individual liver plasmacytoid dendritic cells are capable of producing IFNα and multiple additional cytokines during chronic HCV infection
Source: PLoS Pathog. 2019 Jul 29;15(7):e1007935. doi: 10.1371/journal.ppat.1007935 (PMC6687199; doi:10.1371/journal.ppat.1007935)
Supplement: S5 Table — (DOCX) [file ppat.1007935.s009.docx]

| Supplementary Table 5. CyTOF Panels. | | | |
| --- | --- | --- | --- |
| CyTOF Panel | **Vendor** | **Catalog Number** | **Clone** |
| Anti-human CD11c – In115 | Biolegend | 337202 | Bu15 |
| Anti-human CD33 – Pr141 | Biolegend | 303410 | WM53 |
| Anti-human CD19 – Nd142 | Fluidigm | 3142001B | HIB19 |
| Anti-human CD145RA – Nd143 | Biolegend | 304102 | HI100 |
| Anti-human CD4 – Nd145 | Fluidigm | 3145001B | RPA-T4 |
| Anti-human CD8 – Nd146 | Biolegend | 301002 | RPA-T8 |
| Anti-human IL-1b – Sm147 | Biolegend | 511601 | H1b-27 |
| Anti-human CD16 – Nd148 | Fluidigm | 3148004B | 3G8 |
| Anti-human CD1c – Nd150 | Biolegend | 331502 | L161 |
| Anti-human CD123 – Eu151 | Fluidigm | 3151001B | 6H6 |
| Anti-human CD66b – Sm152 | Biolegend | 305102 | G10F5 |
| Anti-human TNFα – Eu153 | Biolegend | 502941 | MAB11 |
| Anti-human CD86 – Sm154 | Biolegend | 305402 | IT2.2 |
| Anti-human CD27 – Gd155 | Biolegend | 302802 | O323 |
| Anti-human IL-6 – Gd156 | Fluidigm | 3156011B | MQ2-13AS |
| Anti-human CCL3 – Tb159 | R&D Systems | MAB9849 | 93333R |
| Anti-human CD14 – Gd160 | Fluidigm | 3160001B | M5E2 |
| Anti-human CD56 – Dy161 | BD Biosciences | MAB555513 | B159 |
| Anti-human CD64 – Dy162 | Biolegend | 305016 | 10.1 |
| Anti-human CD40 – Dy164 | Biolegend | 334302 | 5C3 |
| Anti-human CCL4 – Ho165 | R&D Systems | MAB271 | 24006 |
| Anti-human IL-10 – Er166 | Biolegend | 501402 | JES3-9D7 |
| Anti-human CD3 – Er168 | Biolegend | 300402 | UCHT1 |
| Anti-human IFNα2b – Tm169 | BD Biosciences | 551795 | 7N4-1 |
| Anti-human CD38 – Er170 | Biolegend | 356602 | HB-7 |
| Anti-human IL-8 – Yb173 | Biolegend | 514602 | BH0814 |
| Anti-human HLADR – Yb174 | Biolegend | 307602 | L243 |
| Anti-human IL29 – Lu175 | R&D Systems | MAB15981 | 247801 |
| Anti-human IP-10 – Yb176 | Biolegend | 519502 | J034D6 |
| Anti-human CD45 – Pt196 | Biolegend | 304002 | HI30 |
| Anti-human CD45 – Pt198 | Biolegend | 304002 | HI30 |
| Anti-human CD11b – Bi209 | Fluidigm | 3209003B | ICRF44 |
